# Recent Advances in Stimuli-Responsive Metallogels

**DOI:** 10.3390/molecules28052274

**Published:** 2023-02-28

**Authors:** Zhixiong Liu, Xiaofang Zhao, Qingkai Chu, Yu Feng

**Affiliations:** 1 School of Chemistry and Chemical Engineering, Shanxi Datong University, Datong 037009, China; 2 School of Materials Science and Engineering, Changzhou University, Changzhou 213164, China

**Keywords:** metallogel, stimuli-responsive, supramolecular chemistry, self-assembly

## Abstract

Recently, stimuli-responsive supramolecular gels have received significant attention because their properties can be modulated through external stimuli such as heat, light, electricity, magnetic fields, mechanical stress, pH, ions, chemicals and enzymes. Among these gels, stimuli-responsive supramolecular metallogels have shown promising applications in material science because of their fascinating redox, optical, electronic and magnetic properties. In this review, research progress on stimuli-responsive supramolecular metallogels in recent years is systematically summarized. According to external stimulus sources, stimuli-responsive supramolecular metallogels, including chemical, physical and multiple stimuli-responsive metallogels, are discussed separately. Moreover, challenges, suggestions and opportunities regarding the development of novel stimuli-responsive metallogels are presented. We believe the knowledge and inspiration gained from this review will deepen the current understanding of stimuli-responsive smart metallogels and encourage more scientists to provide valuable contributions to this topic in the coming decades.

## 1. Introduction

Supramolecular gels, a new class of promising soft materials, have received considerable attention during the last few decades because of their numerous applications in different fields, including supramolecular chemistry, material science, nanoelectronics, catalysis, regenerative medicine, tissue engineering, sensors, drug delivery and environmental science [1,2,3,4,5,6,7,8,9,10,11,12,13,14,15]. In supramolecular gels, low-molecular-weight gelators (LMWGs) or polymers self-assemble via various supramolecular interactions, including hydrogen bonding, π-π stacking, Van der Waals forces, metal coordination, and electrostatic or dipole–dipole interactions, to form the three-dimensional (3D) networks with solvent molecules entrapped inside. Benefiting from the weakness and reversible non-covalent interactions, supramolecular gels are stimuli-responsive, meaning their gel properties and phase transition can be modulated and regulated in response to an external stimulus, such as temperature, light, sound, mechanical force, magnetic fields, electric fields, pH, chemicals and enzymes. Today, stimuli-responsive supramolecular gels are recognized as potential regenerative and self-healing materials, and have exhibited potential for many applications in fields such as of molecular motors, sensors, actuators and drug delivery systems [16,17,18,19,20,21,22,23,24].

Metallogels are a fascinating kind of supramolecular gel wherein metallic elements actively participate in gel formation. Since the discovery of the first metallogel systems based on lithium urate in the mid-19th century, a number of metallogels derived from discrete coordination complexes, coordination polymers and cross-linked coordination polymers, with different chemical structures and functionalities, have been reported to date due to their various potential applications, including sensing, catalysis, magnetism, drug delivery, optoelectronics, nanoparticle templating, environmental remediation and biological systems [25,26,27,28,29,30,31,32,33]. Obviously, the incorporation of metal components is an effective way to subtly tune gelation ability and modify gel morphology via a metal–ligand interaction. Moreover, the addition of metal components is a straightforward way to introduce various interesting and specific properties, including optical, electronic, redox, catalytic and magnetic properties, into the gel matrix. Compared to traditional supramolecular gels, metallogels are able to intelligently respond to a wider range of external physical and chemical stimuli. The weakness of these non-covalent interactions allows the metallogel to collapse or dissolve after being triggered by external stimuli. In addition to the gel–sol phase transition, other changes in the physical characteristics of metallogel systems, such as their color, fluorescence, viscosity, conductivity and magnetic susceptibility, are observed in response to metallogel structural changes. Thus far, various stimuli-responsive metallogel systems with different metal complexes and functionalities have been reported [34,35].

In this review, we outline the state of the art of stimuli-responsive supramolecular metallogels that are constructed from discrete coordination complexes, organometallic compounds or coordination polymers. According to external stimulus sources, we group stimuli-responsive metallogels in three categories: (1) chemical stimuli-responsive metallogels, (2) physical stimuli-responsive metallogels and (3) multiple stimuli-responsive metallogels. Finally, the current challenges and limitations, as well as the potential outlooks for exploiting stimuli-responsive metallogels, are discussed.

## 2. Stimuli-Responsive Metallogels

### 2.1. Chemical Stimuli-Responsive Metallogels

Generally, metal–ligand coordination and noncovalent intermolecular interactions, such as hydrogen bonding, π-π interactions, Van der Waals forces, hydrophobic interactions and metal–metal interactions, are responsible for the gelation process. A delicate balance of these noncovalent intermolecular interactions plays an important role not only in forming a cross-linked network to immobilize solvents on a nanoscale, but also in endowing the resulting gel with stimuli-responsive characteristics. Small structural variation in the metallogelators would result in quite different gelation properties. Therefore, either the addition of competitive ligands (e.g., anions, cations, neutral chemicals, chiral guest molecules, etc.) or oxidant/redox pairs in the metallogel system has a strong tendency to change the coordination mode of the metallogelator and/or the self-assembly process, and thus, results in different gelation behavior. In this section, the chemical stimuli-responsive metallogels were mainly classified into anion-responsive, redox-responsive and chirality-responsive metallogels.

#### 2.1.1. Ion-Responsive Metallogels

Metal-coordinated supramolecular gels show ion-responsive performance due to the preferential electrostatic interaction between metal ions and additive anions. When proper anions are added into metallogel systems, the preferred electrostatic interactions between the anions and the metallic cation of the metallogelator facilitates scavenging of the metal ion from the metallogels. Generally, the loss of the metallic center results in a structural change in the metallogelator and substantially alters the property of self-assembly. 

**Figure 1:**
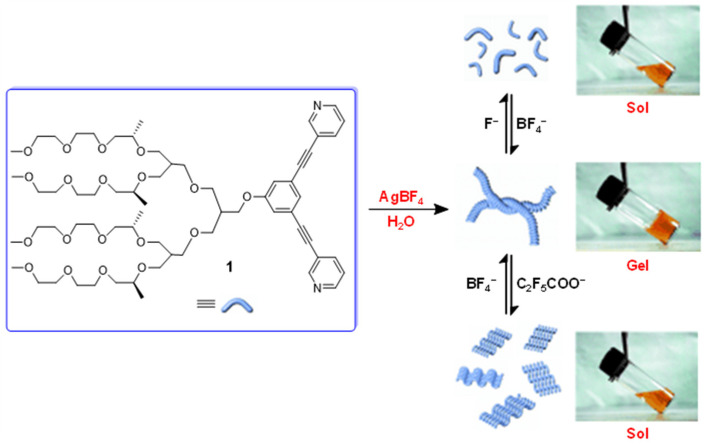
Schematic representation of reversible polymerization and reversible conversion between folded and unfolded conformations of a coordination chain upon counteranion exchange. Reprinted with permission from Ref. [36]. Copyright (2005), John Wiley and Sons.

In 2005, Lee’s group reported a reversible-coordination supramolecular polymer formed from the ligand **1** and Ag(I) ions, which further self-assembled into a helical structure to form an entangled fibrillar network responsible for gelation (Figure 1) [36]. A switchable gel–sol phase transition of this metallohydrogel was observed upon undergoing a counteranion exchange of BF_4_^−^ with F^−^ or C_2_F_5_COO^−^. Its responsiveness was attributed to depolymerization and a conformational change from a folded helical conformation to an unfolded zigzag conformation of the coordination chains.

**Figure 2:**
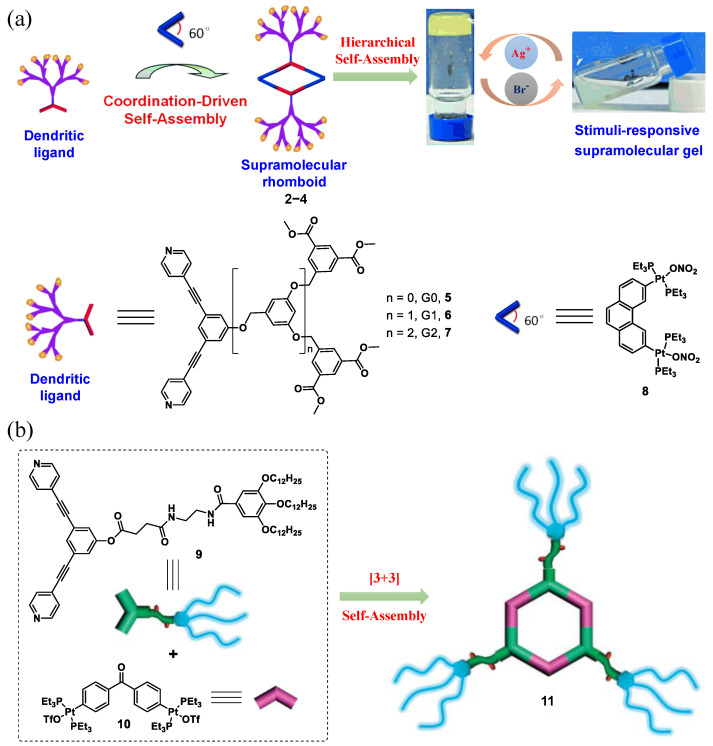
(**a**) Self-assembly of dendritic donors **5**–**7** and acceptors **8** into rhomboids **2**–**4**, and schematic structures of the reversible stimuli-responsive gel–sol phase transition. Reprinted with permission from Ref. [37]. Copyright (2013), John Wiley and Sons. (**b**) Cartoon representation of the formation of hexagonal metallacycle **11** from 120° donor **9** and 120° di-Pt(II) acceptor **10**. Reprinted with permission from Ref. [38]. Copyright (2014), Royal Society of Chemistry.

In 2013, Yang et al. designed and fabricated self-assembled metallodendritic rhomboids **2**–**4** consisting of a peripherally dimethylisophthalate-functionalized poly(benzylether) dendritic ligand **5**–**7** and a diplatinum(II) acceptor **8** [37]. The second-generation metallodendritic rhomboid was found to hierarchically self-assemble into a stable metallogel. The gel was easily broken and finally changed into a turbid solution after the addition of competitive bromide ions. The resulting solution was renewed to form a gel after the addition of AgPF_6_ (Figure 2a). The switchable gel–sol phase transition was accompanied by a morphology change from a fibrous network in the gel state to an irregular microstructure in the sol state. The dynamic metal–ligand coordination was responsible for anion-responsiveness due to the reversible disassembly and reassembly of discrete rhomboidal metallacycles, induced by the preferred interactions between metal ions and bromide. Recently, a discrete hexagonal metallocycle **11**, assembled from a rigid ligand **9** with multiple amide moieties and alkyl chains and a diplatinum(II) acceptor **10**, was developed via [3+3] coordination by Yang’s group (Figure 2b) [38]. A similar gel–sol state transition was observed by alternately adding bromide ions and AgPF_6_ to the gel–sol system.

**Figure 3:**
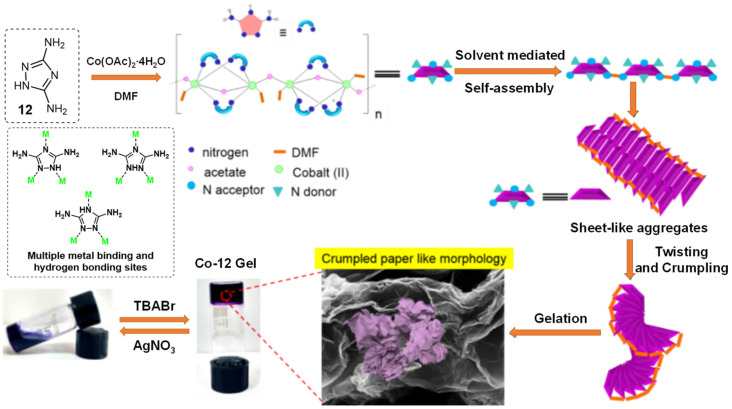
Schematic representation of the probable basic unit of **Co-12** Gel, and the gelation mechanism and its reversible gel–sol transformation towards Br^−^. Reprinted with permission from Ref. [39]. Copyright (2019), American Chemical Society.

Another example of metallogels with bromide-responsiveness was reported by Mitra et al. [39]. The nitrogen-rich triazole ligand **12** was able to form a metallogel cogel upon mixing of a warm solution of 3, 5-diamino-1, 2, 4-triazole (DAT) in dimethylformamide (DMF) with a solution of cobalt acetate (Figure 3). The initial 1D aggregates assembled from the Co(II)-DAT complexes were changed into thin 2D sheets due to the solvent-assisted intermolecular interaction and Co−Co stacking. These 2D sheets were further wrinkled to give 3D crumpled paper sheet-like aggregates, which were responsible for the gelation process. The gel was disturbed, and then, changed into a sol after adding bromide ions. The resulting sol was restored to a gel upon the addition of silver nitrate. The collapse of the gel network was attributed to the presence of a bulky cation and a bromide anion, which weakened the hydrogen-bonded network. Silver nitrate was able to scavenge the bromide anion and form silver halide, which re-established the hydrogen-bonded network and the gel state.

**Figure 4:**
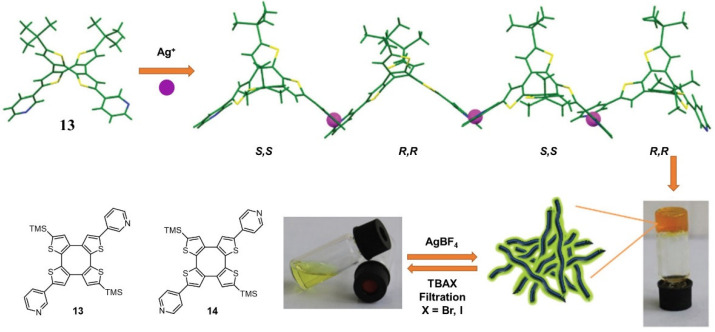
Molecular structure of ligands **13**-**14** and possible mechanism of self-assembly of metallogel **13·**Ag. Reprinted with permission from Ref. [40]. Copyright (2021), Royal Society of Chemistry.

In 2021, a saddle-shaped cyclooctathiophene **13–14** with pyridine groups was synthesized by Wang and coworkers [40]. After coupling with Ag(I) ions, an orange metallogel was formed immediately at room temperature. The formation of the coordination polymer was necessary for gelation. Macroscopic gel–sol transformation was observed after the addition of halogen ions (i.e., Br^−^ and I^−^) because the halides dissolved and transformed into a sol with the precipitation of AgX. The addition of the same amount of AgBF_4_ led to reformation of the metallogel (Figure 4).

**Figure 5:**
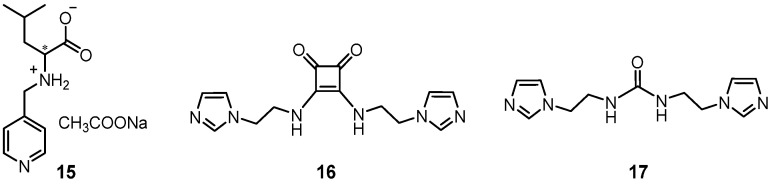
Structures of ligands **15**–**17**.

Two supramolecular metallohydrogels were synthesized from an amino acid-based ligand **15** and zinc/cadmium salts (Figure 5) [41]. These metallohydrogels were observed to be responsive towards chloride and bromide ions, and gradually degraded into different MOF phases spontaneously. Recently, a green opaque metallogel was synthesized via the self-assembly of squaramide-based bisimidazole ligands **16, 17** with copper(II) (Figure 5) [42]. A color change from green to pale purple was observed after adding bromide ions. In contrast, other kinds of anion resulted in a blue metallogel. The color change was also able to occur even when the solid bromide salts were directly added into the gel system. These features made the metallogel system a good platform for the rapid and selective discrimination of bromide from other anions. 

**Figure 6:**
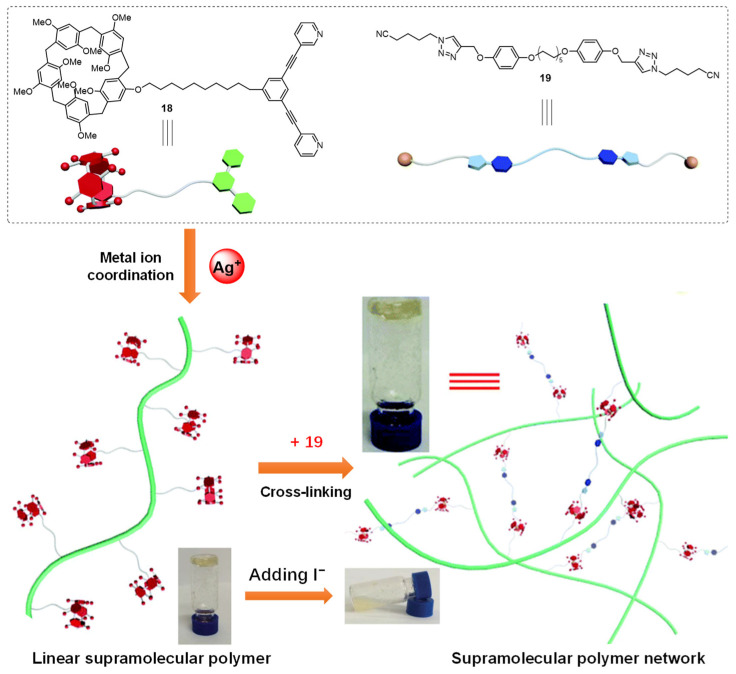
Chemical structures of **18** and **19** and a cartoon representation of the formation of the metallogel. Reprinted with permission from Ref. [43]. Copyright (2017), Royal Society of Chemistry.

In 2017, Shi et al. synthesized a pillar [5] arene-based ligand **18** with two pyridyl groups [43]. A linear supramolecular polymer was firstly prepared based on the coordination-driven self-assembly between a ligand and a silver ion at an accurately equal stoichiometric ratio. A supramolecular polymer network was formed upon the introduction of a ditopic connector **19** containing two triazole binding sites through host–guest recognition between pillar[5]arene and a neutral guest molecule. At a high concentration, the cross-linked polymer network was converted to a stable gel. After adding I^−^ into the prepared metallogel, a gel–sol state transition was observed because the linear polymer was destroyed upon the addition of an iodide anion (Figure 6).

**Figure 7:**
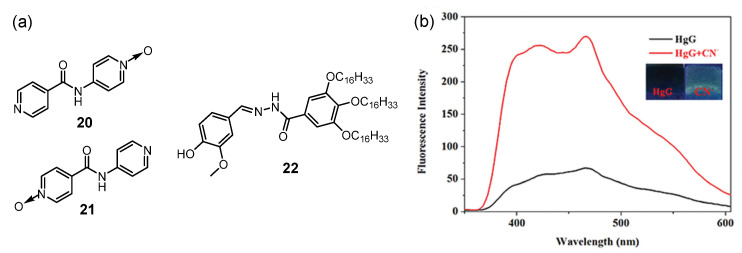
(**a**) Chemical structures of compounds **20**-**22**. (**b**) Change in the emission of the **22**∙Hg^2+^ gel (HgG) in the presence of CN^−^. The inset presents the color change of the **22**∙Hg^2+^ gel (HgG) in the presence of CN^−^. Reprinted with permission from Ref. [46]. Copyright (2019), Royal Society of Chemistry.

Cyanide is widely used in various industrial processes, and the detection of cyanide anions is very important due to their extreme toxicity. A visual gel–sol phase transition that occurred due to the collapse of the metallogel after the addition of cyanide suggests that the anion-responsive metallogel is an ideal platform for the visual detection of cyanide ions [44]. Damodaran et al. reported a supramolecular metallogel from pyridyl-N-oxide (PNO) (**20** and **21**) and Zinc(II)/cadmium(II) with anion stimuli-responsiveness (Figure 7a) [45]. As seen in their XPRD experiment, the crystals and the xerogels of Zn-PNO had a similar crystal phase, suggesting that the structure obtained from the single-crystal data was translated to a hierarchical xerogel network. The zinc(II) metallogel gradually collapsed in the presence of cyanide, fluoride and iodide anions. The cadmium(II) metallogel was able to selectively respond to cyanide anions in water, but was silent in the case of halides. The selective discrimination of cyanide anions was attributed to the stronger coordination of cyanide with cadmium(II), as it scavenged cadmium(II) from the supramolecular metallogel, resulting in disassembly of the polymeric gel network.

**Figure 8:**
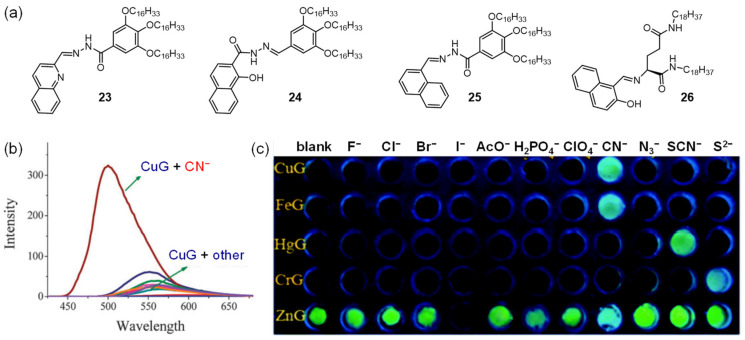
(**a**) Molecular structures of compounds **23**–**26**. (**b**) Fluorescence spectra of CuG (0.8%, in DMF) in the presence of various anions (F^−^, Cl^−^, Br^−^, I^−^, AcO^−^, H_2_PO_4_^−^, N_3_^−^, SCN^−^, ClO_4_^−^, S_2_^−^ and CN^−^) at room temperature. (**c**) Change in the color of different metallogels in the presence of 1 equiv. of various anions (using 0.1 mol L^−1^ anion sodium or potassium salt water solution as anion source). Reprinted with permission from Ref. [48]. Copyright (2015), Royal Society of Chemistry.

Recently, a novel design methodology for competitive coordination control in AIE mode for anion-responsive gels was developed [46]. Moreover, a series of supramolecular metallogel-based anion sensor arrays was designed [47,48,49,50,51]. Ligand **22,** with long-alkyl-chained acylhydrazone, was found to gel DMSO with a low CGC [47]. The emission intensity of the gel at 475 nm increased, suggesting aggregation-induced emission (AIE). With the addition of Hg(II) and Fe(III) to the gel system, the fluorescence emission of the gel was quenched. When CN^−^ was added to the metallogel HgG, the emission intensity at 475 nm was increased (Figure 7b). The detection limit of HgG for CN^−^ was 3.72 × 10^−9^ M. Subsequently, Lin et al. designed a family of organogelators (**23** [48], **24** [49] and **25** [50]) with an acylhydrazone functionality (Figure 8a), which showed strong AIE in the organogel state. After adding metal ions such as Cu(II), Hg(II), Fe(III) or Cr(III) into the organogel system, the AIE of the organogel was quenched and corresponding nonfluorescent metallogels were formed. The resulting metallogel, Cu-**23**, was able to selectively sense cyanide anions via fluorescence “turn on” (Figure 8b, c). With the diffusion of the cyanide anions, the competitive coordination between the cyanide and metal ions scavenged the metal ions from the metallogel system, resulting in fluorescence “turn on”. Powder-XRD experiments further proved the presumed mechanism. After adding Cu(Ⅱ) ions, a new strong peak at 2*θ* = 22.12°, corresponding to *d*-spacing of 3.84 Å, was observed, which was attributed to the coordination of Cu^2+^ with organogelator **23**. This strong peak disappeared after the addition of cyanide anions, suggesting that the cyanide anions competitively bound to Cu^2+^ and the complex was disassembled. An L-glutamic acid Schiff base derivative **26** was synthesized (Figure 8a) [51]. After coupling with metal ions, the formed metallogel could be used to detect CN^−^ by monitoring their fluorescence.

#### 2.1.2. Redox-Responsive Metallogels

In addition to ions, the supramolecular metallogels also showed responses to outer oxidant/redox reagents or electric field stimuli [52]. The redox reaction led to structural variation in the metallogelator and a change in the self-assembly mode of the metallogelator, which finally resulted in a sol–gel transition, a color change, weakened/enhanced fluorescence, etc. These redox-responsive metallogel systems have attracted increasing interests due to their potential applications in drug delivery, sensors, the storage of energies, etc.

**Figure 9:**
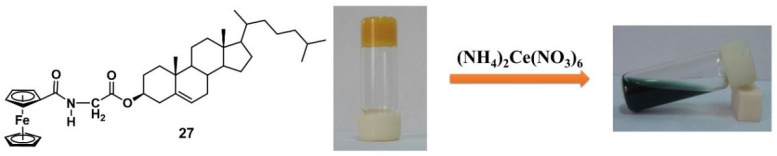
Chemical structure of the gelator **27** and responsiveness of the ethyl acetate gel of compound **27** to the oxidant (NH_4_)_2_Ce(NO_3_)_6_. Reprinted with permission from Ref. [56]. Copyright (2008), Elsevier.

The ferrocenyl group, as a redox center, can be easily oxidated to form a cation state in the presence of an oxidant, and has been widely used to construct redox-responsive metallogels [53,54,55]. In 2008, Fang’s group reported a cholesterol-appended ferrocene derivative **27,** and the metallogelator was able to form a stable orange metallogel in cyclohexane [56]. When the proper amount of oxidant (NH_4_)_2_Ce(NO_3_)_6_ was placed into the metallogel, the gel gradually became a dark-green suspension (Figure 9). The resulting suspension was converted to a stable orange gel upon treatment with a reductant such as hydrazine. The switchable gel–sol transition was caused by alternately conducting chemical oxidation and the reduction of ferrocenyl moieties.

**Figure 10:**
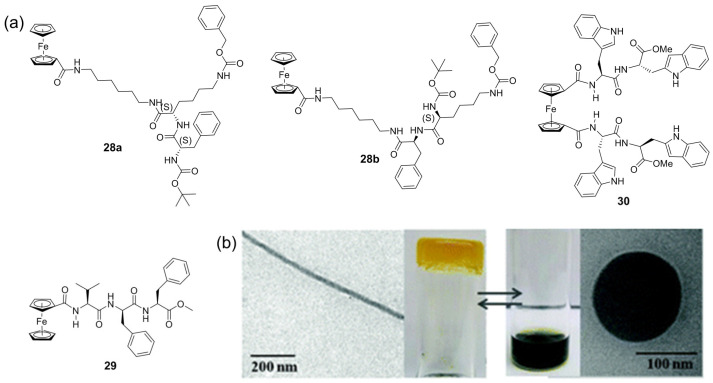
(**a**) Chemical structure of the metallogelator **28**–**30**. (**b**) Schematic representation of redox induced morphological transformations of Fc-peptide **29** via different modes of assembly. Reprinted with permission from Ref. [59]. Copyright (2014), Royal Society of Chemistry.

Kraatz et al. designed and synthesized a family of novel ferrocene–dipeptide conjugates **28–30**, which are capable of forming metallogels in response to redox stimuli [57,58,59,60]. These metallogelators **29** were able to form a stable metallogel in toluene after a short period of ultrasonication irradiation [59]. The gel was gradually degraded after the addition of the oxidizing agent Fe(ClO_4_)_3_. The resulting deep-blue solution was changed into a stable metallogel after treatment with ascorbic acid as a reductant. The switchable gel–sol phase transition was attributed to a reversible change between ferrocene and ferrocenium, which was triggered by alternately treating with Fe(ClO_4_)_3_ and ascorbic acid. A morphological change from a fibrillar network in the gel state to micelles in the sol state was observed during the redox-responsive process (Figure 10b).

**Figure 11:**
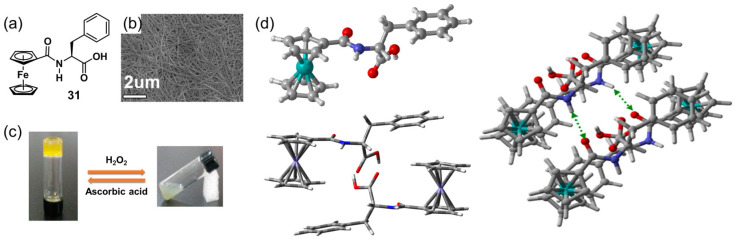
(**a**) Chemical structure of the gelator **31**. (**b**) SEM image of the cryo-dried hydrogel **31**. (**c**) Reversible gel−sol transitions of the supramolecular hydrogel triggered by chemical redox reaction. (**d**) Ab initio calculation of the self-assembly pathway of the Fc-F monomer. The red bar with a white end represents an −OH group, and the bar with a red end is the carbonyl group. Reprinted with permission from Ref. [61]. Copyright (2013), American Chemical Society.

Zhang et al. designed a ferrocenoyl phenylalanine metallogelator **31** [61], which was able to form a yellow metallohydrogel. DFT calculation indicated that the hydrogel formation involved an antiparallel, noncovalent dimerization step wherein the ferrocenoyl functionalities of one axe-like monomer became conjoined with the phenyl moiety of the second monomer via a π–π interaction to form a brick-like dimer (Figure 11d). The formed hydrogel showed effective chemical and electrochemical redox-responsiveness. When an aqueous H_2_O_2_ solution was carefully added to the top of the metallogel, the gel gradually collapsed and finally became a light-green suspension. A small amount of reductant ascorbic acid solution induced the suspension to reform the gel state after adjustment of the pH value (Figure 11c). A reversible morphology change between the nanofibers (Figure 11b) and the amorphous structure was observed during the response processes. A chemical structure change from the neutral ferrocenoyl group to ferrocinium ions was responsible for the gel–sol state transition. Switching between the gel state and the sol phase as a function of redox potential was successfully realized by carefully designing a proper electrode area and ensuring correct gel film thickness.

**Figure 12:**
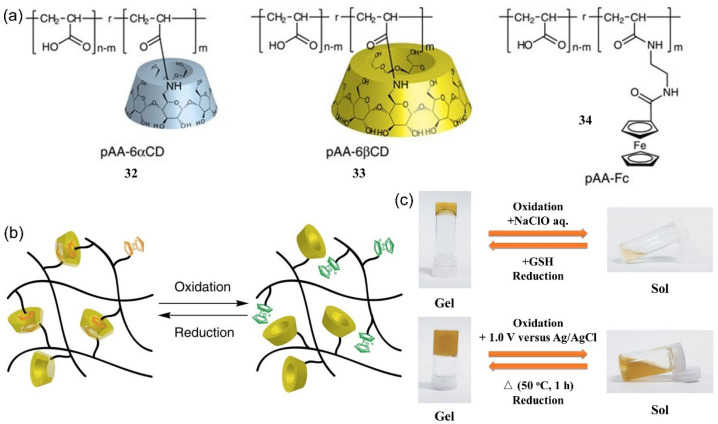
(**a**) Chemical structures of the host polymers **32** and **33** and the guest polymer **34**. (**b**) Schematic illustration of sol−gel transition. (**c**) Sol−gel transition experiment using chemical reagents and electrochemical reactions. Adding NaClO aq. to the **33**/**34** hydrogel induced a phase transition into the sol state, and continuous addition of GSH to the sol recovered the elasticity to yield the hydrogel again. Electrochemical oxidation (+1.0 V versus Ag/AgCl) transformed the hydrogel into sol, whereas reduction recovered the viscosity, reverting it back to a hydrogel. Reprinted with permission from Ref. [62]. Copyright (2012), Nature Publishing Group.

In 2011, Harada et al. developed a redox-responsive supramolecular metallohydrogel by inducing a host–guest interaction, using poly(acrylic acid) modified with cyclodextrins (PAA-CDs) **32**-**33** as a host polymer and PAA possessing ferrocene (PAA-Fc) **34** as a guest polymer (Figure 12a) [62]. The metallohydrogel underwent a gel–sol phase transition upon using NaClO as an oxidant. The sol could be successfully restored to a gel state after treatment with the reductant glutathione (GSH) (Figure 12c). The response mechanism was probably due to the change in the host–guest interaction. β-cyclodextrins (β-CD) had a higher affinity for the reduced state (Fc) compared with the oxidized state (Fc^+^) of the ferrocene. After treatment with NaClO, the neutral ferrocenoyl group was oxidized to yield ferrocinium ions, resulting in dissociation of the host–guest complexes responsible for the gel–sol state change (Figure 12b). In addition to oxidant/redox reagents, an electric field stimulus also triggered a gel–sol phase transition (Figure 12c). Electrochemical oxidation of the metallohydrogel triggered the gel–sol transformation. Subsequent reduction by heating the sol at 50 °C restored the gel state.

**Figure 13:**
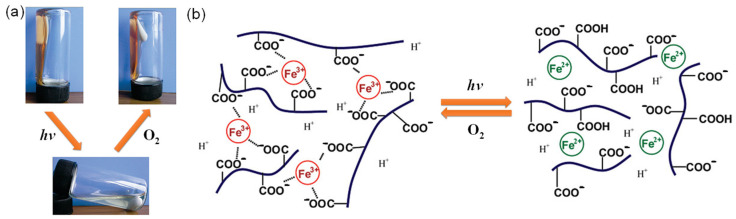
(**a**) Switching gel−sol−gel transition in PAA (20 wt%) + Fe(III) (0.02 mol/L)−citrate (0.04 mol/L) aqueous system at pH 4.0 and room temperature. (**b**) Schematic illustration of the gel−sol transition in the PAA + Fe(III)−citrate aqueous system switched via photoreduction and oxidation. Reprinted with permission from Ref. [63]. Copyright (2008), American Chemical Society.

In addition to ferrocenyl groups, a switchable change in Fe(III) and Fe(II) has also been used to fabricate redox-responsive metallogels. Tong et al. developed a redox-responsive gel–sol phase transition metallogel with a poly(acrylic acid) (PAA) aqueous solution containing a Fe(III)-citrate salts, induced by the redox state of ferric ions conjugated with photoreduction and oxidation [63]. A homogeneous metallohydrogel was immediately formed after adding trivalent cations, such as Fe(III) ions, into a PAA solution due to the strong binding between trivalent cations and the carboxyl groups. The metallogel was transformed into a colorless solution upon irradiation with sunlight for a few minutes (Figure 13a). The metallogel dissolution was attributed to the photoreduction of Fe(III) to Fe(II) ions in the presence of reductive citric acid under sunlight (Figure 13b). The resulting solution was restored to a homogeneous gel by oxidating Fe(II) to Fe(III) with oxygen. This gel–sol phase transition triggered by sunlight irradiation and oxygen can be repeatedly carried out. Subsequently, Xu et al. created a redox-responsive metallohydrogel by mixing polyvinyl alcohol acetoacetate (PVAA) aqueous solution and Fe(III) aqueous solution through dynamic and reversible supramolecular complexation [64]. The gel state was converted to a colorless solution upon irradiation with ultraviolet light for a few minutes due to the photoreduction of Fe(III) to Fe(II) ions. A homogeneous brown gel was successfully reconstructed either after being exposed to oxygen or upon the addition of excessive H_2_O_2_.

**Figure 14:**
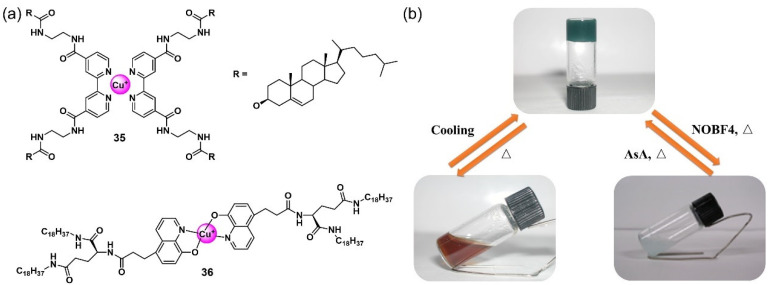
(**a**) Chemical structures of the gelators **35** and **36**. (**b**) Photographs of the chromatic change and phase transition behavior of 1-butyronitrile (1-PrCN) gel of **35**. Reprinted with permission from Ref. [65]. Copyright (2004), American Chemical Society.

A switchable state change between Cu(II) and Cu(I) in the metallogelator has also been used to develop redox-responsive metallogel systems [65,66,67,68]. Shinka et al. created the first redox-responsive supramolecular metallogelator **35** using the redox-active Cu(II)/Cu(I) via coordination chemistry (Figure 14a) [65]. A coordination of 2, 2-bipyridine derivative containing two cholesteryl moieties with a Cu(II) ion was able to form stable metallogels in some organic solvents following a heating–cooling process. The formed deep-green metallogel was changed into a sol with a small amount of pale-blue precipitate after being added into NOBF_4_. The gel state was successfully restored after being reduced by ascorbic acid, followed by a heating–cooling process (Figure 14b). A change between the oxidized and reduced state of copper ions was responsible for the gel’s redox-responsive performance. Liu’s group developed a Cu(II) complex of quinolinol-substituted L-glutamide **36** with redox-responsiveness [66]. The complex formed a dark-red metallogel in the THF due to strong hydrogen-bonding interactions and π–π stacking of the aromatic rings, which was enhanced by the coordination mode. The formed dark-red metallogel was turned into a transparent yellow sol upon the addition of a reductant such as ascorbic acid. Subsequently, the sol was treated with a flow of O_2_ for 30 min, and a stable dark-red metallogel was reformed. The reversible gel–sol phase transition could be repeated many times. During the redox-responsive process, the morphology of the assembly also changed from nanofibers in the gel state to a flat configuration in the sol state.

**Figure 15:**
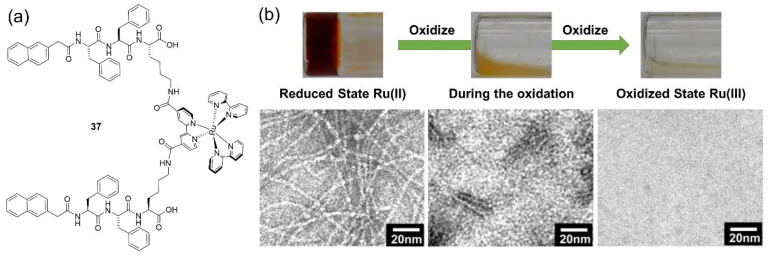
(**a**) Molecular structures of the metallohydrogelator **37**. (**b**) Optical images of oxidation-induced gel−sol transition and TEM images corresponding to the samples at different states of transition. The hydrogel (reduced state) was formed by 0.8% (*w*/*v*) **37** in water at pH = 1. Reprinted with permission from Ref. [69]. Copyright (2013), American Chemical Society.

Metallogelators with other metallic centers, including Ru(II) [69], Co(II) [70,71,72] and Ag(I) [73] ions, have also been synthesized to develop redox-responsive metallogels. Xu et al. designed a ruthenium(II)/tris(bipyridine) complex **37**, and this metallohydrogelator was found to self-assemble in water to form a supramolecular hydrogel [69]. The self-assembled nanofiber in water had a similar width to the metallohydrogelator. The hydrogel preserved the redox property of a polypyridyl ruthenium complex. After adding oxidant Ce(SO_4_)_2_ onto the top of the hydrogel, the hydrogel was changed into a yellow emulsion and finally transformed into a transparent solution. This gel–sol phase transition was caused by the oxidation of Ru(II) to Ru(III) ions. The 3D network, consisting of entangled long and flexible nanofibers, was also gradually dissociated in the responsive process (Figure 15b). Metallo-supramolecular gels consisting of a multitopic cyclam bis-terpyridine platform (CHTT) and cobalt(II) ions were reported and found to be electrochemically and reversibly commuted between gel (red) and liquid (green) states [70]. A novel supramolecular complexation between native CS chains and various metal ions, including Ag(I), Cu(II), Co(II), Ni(II), Zn(II), Cd(II) and Pd(II) ions, resulted in a fast hydrogelation process [73]. The CS-Ag hydrogel was able to respond to various chemical redox stimuli. An aqueous H_2_O_2_ solution was carefully placed onto the top of the CS-Ag hydrogel, which induced the hydrogel to gradually dissociate, and finally, become a clear solution. However, this responsive performance was irreversible.

#### 2.1.3. Chirality-Responsive Metallogels

**Figure 16:**
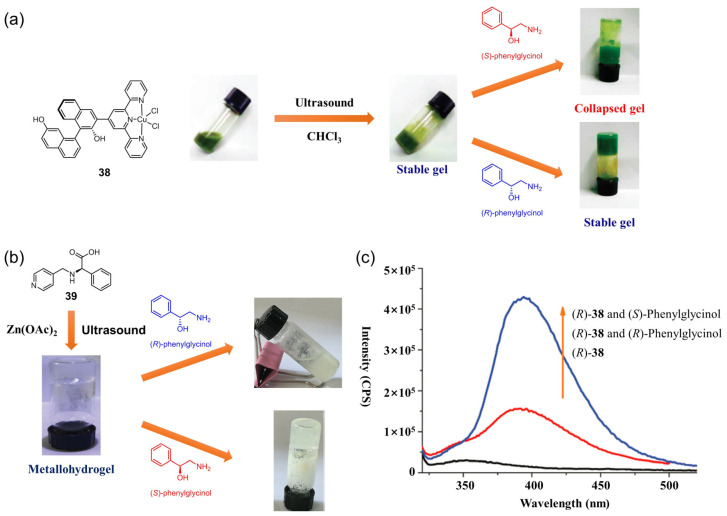
(**a**) Photographs of the enantioselective responses of the gel of (*R*)-**38** toward (*R*)-phenylglycinol and (*S*)-phenylglycinol. Reprinted with permission from Ref. [74]. Copyright (2010), American Chemical Society. (**b**) Enantioselective responses of the metallohydrogel toward (*R*)-phenylglycinol and (*S*)-phenylglycinol. (**c**) Fluorescence spectra of (*R*)-**38** (5.0 × 10^−7^ M) in CH_2_Cl_2_/nhexane (2:3) in the presence of (*R*)- and (*S*)-phenylglycinol (5.0 × 10^−4^ M) (*λ*_exc_ = 289 nm). Reprinted with permission from Ref. [75]. Copyright (2020), Royal Society of Chemistry.

The development of a simple and facile method to distinguish left- and right-handed enantiomers is intriguing and would be useful in pharmacology and biology, but it is still a big challenge. Supramolecular gels are considered a useful platform for the visual discrimination of chiral enantiomers due to their unique stimuli-responsiveness [10]. A variety of complexes consisting of chiral ligands and metallic centers can form stable metallogels in various solvents and have shown interesting chiral effects. A visual gel–sol phase transition resulting from a conformation change in the metallogelator due to a competitive coordination effect between the preferred enantiomer and the metallic center could be used to visually discriminate the chiral enantiomer. In 2010, Pu et al. reported the first example of chiral enantiomer-responsive metallogels containing a BINOL–terpyridine-based ligand and a Cu(II) ion [74]. The (*R*)-BINOL–terpyridine-based Cu(II) complex **38** formed an opaque-green metallogel in CH_3_Cl after sonication for a few minutes. When (*S*)-phenylglycinol (0.1 equiv) was added to the above metallogel, the metallogel gradually collapsed. However, the addition of the same amount of (*R*)-phenylglycinol had little effect on the metallogel, and thus, did not result in a gel–sol phase transition (Figure 16a). This phenomenon suggested an enantioselective nature of the metallogel’s response toward chiral amino alcohol. The fluorescence responses of **38** toward (R)- and (S)-phenylglycinol in solution were further investigated. The fluorescence of the BINOL–terpyridine ligand had a strong emission at λ = 396 nm. The emission was almost completely quenched in the complex **38.** The emission at 396 nm was greatly enhanced after the addition of (S)-phenylglycinol. However, the fluorescence enhancement was much weaker when the complex **38** was treated with (R)-phenylglycinol (Figure 16c). The enantioselectivity was attributed to the enantioselective scavenging of the Cu(II) ion from the metallogel, because the coordination between the metallogelator and (*S*)-phenylglycinol was more favorable than the reaction with (*R*)-phenylglycinol. Recently, Liu et al. designed a chiral metallohydrogel based on a phenyl-((pyridin-4-ylmethyl)-amino)-acetic acid **39** and Zn(II) ion [75]. The as-synthesized metallohydrogel acted as a visual chiral sensor for discriminating between (*R*)-phenylglycinol and (*S*)-phenylglycinol (Figure 16b).

**Figure 17:**
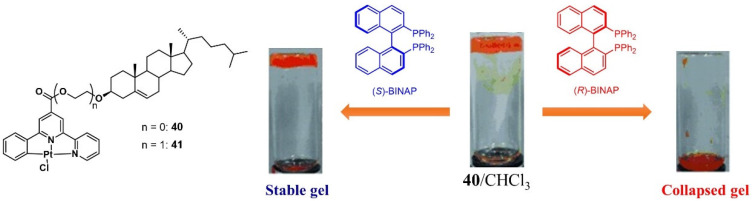
Visual chiral recognition of (*R*)- and (*S*)-BINAP through gel **40**/CHCl_3_ (1 wt%) enantioselective collapsing. Reprinted with permission from Ref. [76]. Copyright (2011), John Wiley and Sons.

In 2011, Tu et al. designed an ALS metallogelator **40**-**41** consisting of a chiral steroidal skeleton and a platinum pincer metallacycle [76]. The metallogelator was able to form stable metallogels in various solvents, driven by Van der Waals interactions, π stacking and metal–metal bonding. When (*R*)-BINAP (0.1 eqiuv) was added into the prepared metallogel **40**/CHCl_3_, the metallogel underwent a gel–sol phase transition. However, the same amount of (*S*)-BINAP could not trigger a gel–sol phase transition even after a heating–cooling process (Figure 17). The (*R*)-BINAP, as a matched chiral guest, was able to coordinate with the Pt center, and thus, the π stacking and metal–metal bonding were broken due to the hindrance of the bulky BINAP skeleton and the PPh_2_ group. Disassembly of the gel network was observed. The other enantiomer, as an unmatched chiral guest, had little impact on the assembly process.

### 2.2. Physical Stimuli-Responsive Metallogels

Physical stimuli, such as heat, mechanical forces and ultrasound irradiation, were also used as triggers to induce an imbalance in a self-assembly system in an unstable state, which would be partly or wholly different from the initial state. Therefore, physical stimuli, including heat, light, mechanical force and ultrasound irradiation, can trigger a switchable property change in metallogels. Unlike the chemical stimulus, the physical stimulus can be applied remotely and non-invasively, and thus, the metallogel system could repeatedly respond to physical stimuli without contamination or degradation of the initial metallogel. A variety of physical stimuli-responsive metallogels have been reported [19,20,21]. Among the various physical stimuli, sonication and mechanical stress have been recognized as important stimuli to control the gel-forming process and gel-responsive behavior. However, it is still challenging to precisely design a metallogel that can respond to sonication and/or mechanical force. A large number of physical stimuli-responsive metallogels have been discovered accidentally. In this section, we mainly focus on ultrasound- and shear-responsive (namely thixotropic) metallogels.

#### 2.2.1. Ultrasound-Responsive Metallogels

Ultrasound irradiation is a type of high-frequency mechanical wave used to support propagation [77]. When a medium such as liquid or suspension is treated with ultrasound irradiation, a huge number of cavities with high energy are formed. The high-energy cavitation process can cause certain chemical and physical changes in the physical medium. It is intriguing that ultrasound has been observed to promote the gelation process. Sonication-induced gel formation is attributed to a rapid crystallization process that facilitates the formation of one-dimensional fiber-like aggregates in a metastable system. Sonication-driven gel formation can occur at room temperature without a heating–cooling process, which gives these metallogels application potential [78,79].

**Figure 18:**
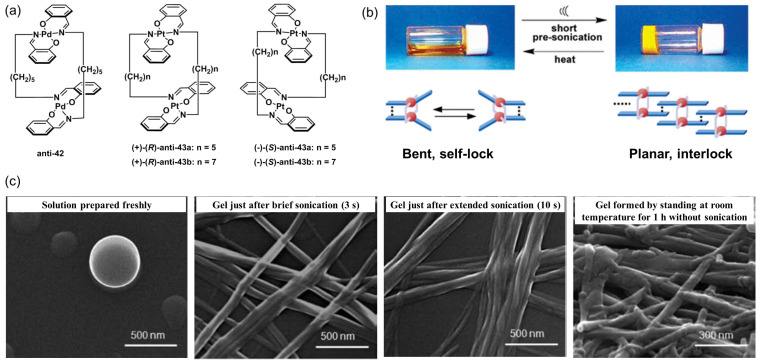
(**a**) Pd- and Pt-Salicylidene complexes **42** and **43** evaluated in sonication-induced gelation. (**b**) Ultrasound-induced gelation of **42** with schematic molecular packing. (**c**) SEM images of dried (()-anti-**43a** aggregates prepared from a 1.50 × 10^−3^ M solution in cyclohexane using various methods. Reprinted with permission from Refs. [81,82]. Copyright (2005, 2011), American Chemical Society.

In 2005, a sonication-induced metallogel formed by a dinuclear Pd complex, **anti-42,** was reported for the first time by Naota and coworkers [80] (Figure 18a). A homogeneous solution of **anti-42** in various organic solvents, such as acetone, CCl_4_, 1,4-dioxane and ethyl acetate, was irradiated via ultrasound (0.45 W/cm^2^, 40 kHz) for a few seconds to provide opaque metallogels. The sonication-induced gelation was attributed to a conformational change from a clothespin-like, bent conformation to a self-lock–interlock structure (Figure 18b). The prepared metallogel was converted to its original sol state following a heating–cooling process. The sol–gel transition could be repeatedly conducted for many cycles. Subsequently, the authors developed another ultrasound-induced metallogel with phosphorescent emission by replacing the palladium ion of **42** with a platinum ion [81]. Non-emissive homogeneous solutions of racemic **anti-43a** with a short alkyl chain (*n = 5*), and optically pure **anti-43b** with a long alkyl chain (*n = 7*), in various organic liquids, were converted immediately into stable phosphorescent metallogels when irradiated using low-power ultrasound (40 kHz, 0.45 W/cm^2^) for a few seconds. The morphological change in the **anti-43a** aggregates was examined via SEM. Spherical nanoparticles with a ~400 nm diameter were observed for the solution of anti-**43a** in cyclohexane. Highly regulated, long and thin fibers with a ~50 nm width were formed after ultrasonic irradiation for 3 s. The prolonged sonication resulted in a high-order nanostructure with flat bundles of fiber units. Irregular, worm-like aggregates were spontaneously formed on the metallogel upon standing at room temperature without sonication (Figure 18c). Strong phosphorescent emission was observed exclusively after ultrasound-induced gelation. The phosphorescent emissive properties of the metallogels could be finely tuned by changing the sonication time, linker length, and optical activity of the metallogelator. Structure-dependent homo- and heterochiral aggregations were responsible for the emission enhancement.

**Figure 19:**
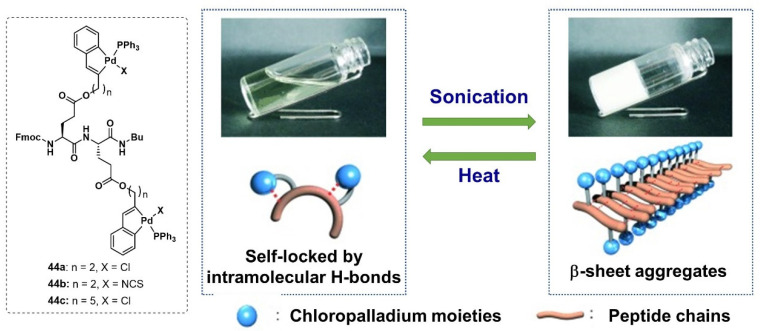
The molecular structure of metalated peptides **44a** and reversible gelation of a 1.50 × 10^−2^ M solution of **44a** in EtOAc at 25 °C. The solution is shown before and just after sonication (0.45 W cm^−2^, 40.0 kHz, 60 s). Reprinted with permission from Ref. [82]. Copyright (2007), John Wiley and Sons.

Later, the authors designed another palladium complex **44a-c** with a peptide linkage [82]. This complex **44a** could gel ethyl acetate c and chlorobenzene under ultrasound (0.45 W/cm^2^, 40 kHz) treatment. The formed metallogel was turned into a sol state via a heat and cooling process. Before sonication irradiation, the intramolecular H-bonding involving the chloro ligand **44a** prevented the dipeptide from undergoing intermolecular self-assembly via H-bonding. Ultrasound treatment released the self-lock and resulted in semistale initial aggregates (Figure 19). The initial domain spontaneously formed β-sheet aggregation with free excess complexes. The content of this active domain increased with the duration of sonication, causing an accelerated gelation rate and higher-order nanostructures with good heat resistance properties.

**Figure 20:**
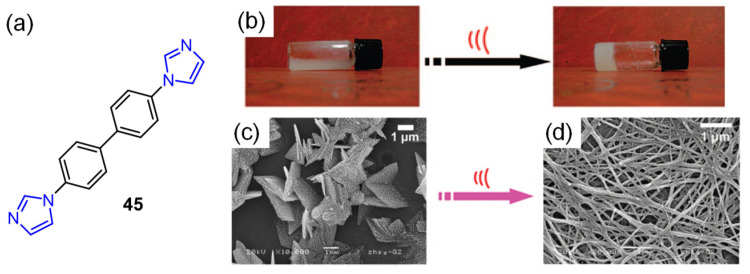
(**a**) Chemical structure of the ligand **45**; (**b**) coordination polymer {Zn(**45**)_2_(OTf)_2_}n in MeOH: the suspension before sonication (left), and the gel after sonication (right); (**c**) sheet-like microparticles of {Zn(**45**)_2_(OTf)_2_}n; (**d**) SEM image of the xerogel. Reprinted with permission from Ref. [83]. Copyright (2009), American Chemical Society.

In 2009, You et al. reported mixing a methanol solution of 4,4′-bisimidazolylbiphenyl **45** with a methanol solution of Zn(OTf)_2_ at room temperature, which resulted in a white suspension [83]. However, the suspension was irradiated via sonication (0.45 W/cm^2^, 40 KHz) for a few minutes, and complete and homogeneous liquid gelation was observed (Figure 20b). In addition, a morphological change from sheet-like coordination polymer microparticles (Figure 20c) into nanofibers (Figure 20d) in the gel state was observed due to the breakage and reorganization of coordination bonds, triggered by sonication. Sonication-induced gelation was believed to originate from the change in the coordination chemistry of Zn(II) ions from tetrahedral to seesaw geometry.

**Figure 21:**
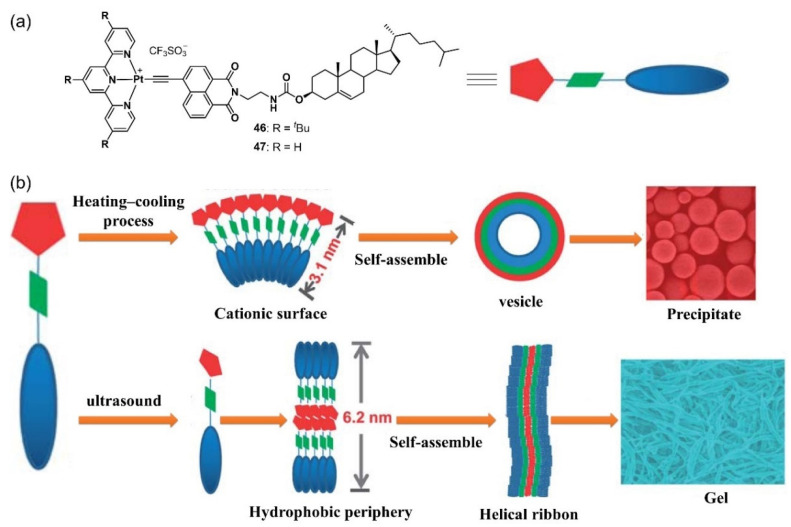
(**a**) The molecular structure of the gelators **46** and **47**. (**b**) A schematic of the mechanism of gelation via a self-assembly process in the sonicated gel **46**. Reprinted with permission from Ref. [84]. Copyright (2013), Royal Society of Chemistry.

In 2013, Yi et al. designed two terpyridyl platinum-based metallogelators **46**-**47** containing hydrophobic cholesterol moiety (Figure 21a) [84]. The metallogelator **46** with three bulky *tert*-butyl groups was able to gel various organic solvents, including ethyl acetate, toluene and *n*-propanol, under sonication irradiation conditions. However, a precipitate was observed following a heating–cooling process. In the thermodynamic process, a single-layered structure self-assembled via hydrophobic interactions aggregated and curved to form a vesicle. Sonication irradiation triggered a molecular conformation change and promoted an ionic dipolar interaction and hydrophobic interactions, and thus, caused the structure to transform into a double-layered structure with the cholesterol groups composing the periphery of the nanoribbons; this finally cross-linked to form a 3D network that was responsible for the gelation process (Figure 21b). The sonication-induced metallogel in *n*-propanol showed different colors (from yellow to red) and photoluminescence enhancement due to the AIE effect. The morphology and surface wettability could be reversibly adjusted via alternation between sonication and heating.

**Figure 22:**
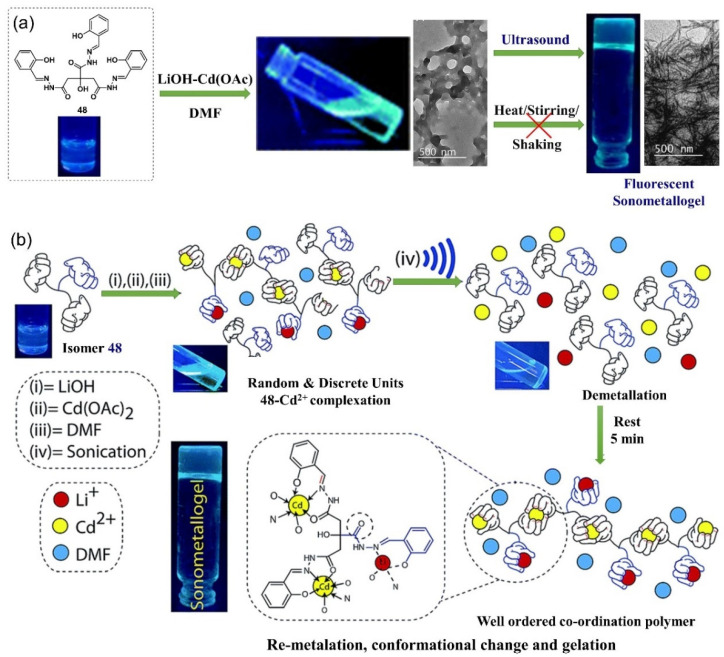
(**a**) The chemical structure of the ligand **48** and the photographs and morphologies of the formed metallogel. (**b**) Model depiction of steps involved in sonometallogel formation along with the binding mode of the ligand **48**. Reprinted with permission from Ref. [85]. Copyright (2017), Royal Society of Chemistry.

In 2017, Dubey et al. reported a novel ultrasound-induced fluorescent metallogel with conducting properties, produced from the non-fluorescent citric acid-derived ligand **48**, LiOH and Cd(OAc)_2_ [85]. A fluorescent transparent metallogel was formed from a mixture of the ligand **48**, LiOH and Cd(OAc)_2_ in DMF via sonication irradiation (16 W, 33 ± 3 kHz) (Figure 22a). However, the traditional methods, including heating, shaking or stirring the mixture of reactants in solution, could not provide a stable metallogel. The morphology of the diluted ultrasound-induced metallogel showed a three-dimensional network of cross-linked nanofibers with ∼20 nm diameters and measuring several micrometers in length. Conversely, random non-directional aggregates were observed under the same conditions before sonication. After mixing, each Cd(II) center was coordinated with two hydrazone-containing ONO-binding arms from two different ligands to yield a random coordination metallopolymer. After sonication treatment, ultrasound irradiation first facilitated the de-metallation process through disruption of the dynamic metal–ligand interaction. Then, suitable conformational changes in the ligand and reformation of the coordination bond provided a well-ordered coordination polymer that was responsible for the gelation process (Figure 22b). In addition, the sonication-induced metallogel showed better ionic conductivity than the unsonicated mixture.

**Figure 23:**
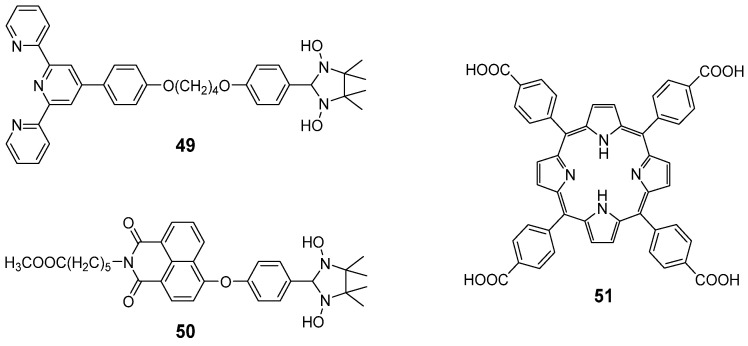
Chemical structures of **49**–**51**.

Recently, Yu et al. developed a novel ligand **49** functionalized with hydroxylamine units and terpyridine [86]. The ligand could selectively gel with copper ions in water via sonication treatment. The sonication-induced metallogel showed a fiber cross-linked morphology, which was different from the flowerlike structure of the metallogel formed via a heating–cooling treatment. Sonication irradiation also triggered a color change from green to blue due to the formed nitroxide radicals. These metallogels were further used as catalysts for the click reaction in water. Another sonication-induced conductive metallogel was developed from a similar ligand **50** with alkali ions [87]. A homogeneous solution of **51** in the presence of Pd(II) in acetonitrile was irradiated with ultrasound for a few seconds to give an opaque metallogel (Figure 23) [88].

#### 2.2.2. Thixotropic Metallogels

Physical shear stress, as a weaker mechanical force, can also be used to tune gel properties. Most supramolecular gels can undergo gel–sol phase transition after stimulation with mechanical stress, and the gel state can be reestablished via a heating–cooling process. Interestingly, some of these mechano-responsive metallogels show the ability to regain their consistency in situ after the removal of the shear stress; these are referred to as thixotropic metallogels. Therefore, these thixotropic metallogels have attracted much interest, as they could be developed into autonomously and intrinsically self-healing materials capable of healing damage simply through the dynamic bonding in the metallogels’ systems.

**Figure 24:**
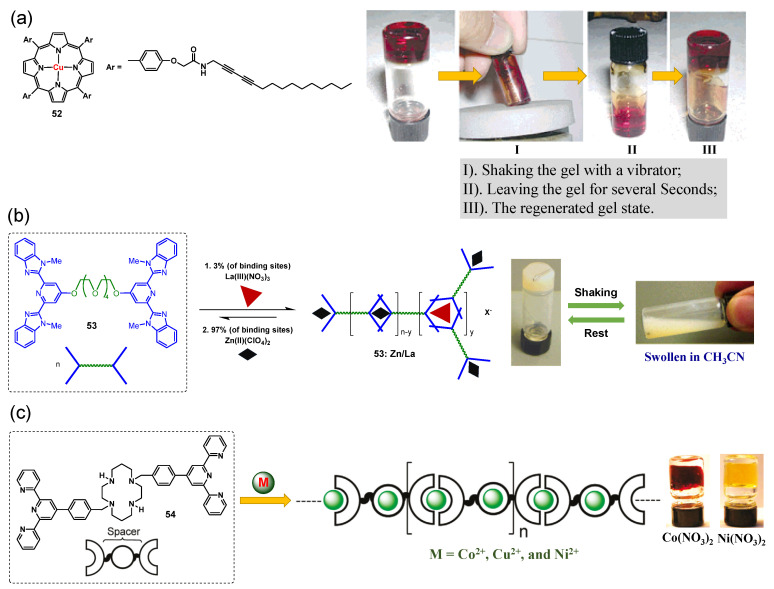
(**a**) Photographs showing the thixotropic behavior of the decalin gel of **52** ((**52**) = 10 g dm^−3^). Shaking the gel with a vibrator resulting a gel-to-sol phase transition (I, II). The regenerated gel state after resting (III). Reprinted with permission from Ref. [89]. Copyright (2005), American Chemical Society. (**b**) The mechano-responsive nature of the thixotropic **53**:Zn/La system. Both materials are swollen in acetonitrile (800% by wt.). Reprinted with permission from Ref. [90]. Copyright (2003), American Chemical Society. (**c**) Structure of the ligand **54** and schematic representation of the formation of the metallopolymers. Reprinted with permission from Ref. [73]. Copyright (2009), American Chemical Society.

In 2005, Shinkai et al. designed an alkylamide group-modified tetraphenyl porphyrin **52** and the metallogelator was able to form a transparent metallogel in cyclohexane and decalin [89]. The metallogel in decalin was observed to be thixotropic (Figure 24a). Rowan and coworkers designed a ditopic ligand **53** consisting of a 2, 6-bis-(1′-methylbenzimidazolyl)-4-oxypyridine unit attached to either end of a penta(ethylene glycol) core [90,91]. The ditopic ligand **53** was polymerized to form a stable metallogel in acetonitrile in the presence of Zn(II), along with a small percentage of La(III), through metal–ligand coordination. The formed metallogel showed pronounced thixotropic performance. Shaking the metallogel vigorously resulted in the formation of a free-flowing liquid, which reformed into a turbid white metallogel upon standing (Figure 24b). This thixotropic behavior was quantitatively evaluated via a shear stress loop test. Yield stress was estimated via direct extrapolation of the straight line portion of the curve to the stress axis, σ_y_ =180 ± 50 Pa. A limited amount of flow occurred when the yield stress was smaller than this critical stress value (I). The metallogel thinned catastrophically once the yield stress increased beyond the critical value (II). Upon reversing the stress, the sol state remained until the stress fell below a comparatively low value (~25 Pa), at which point the gel appeared to begin to be restored. In 2009, Terech et al. reported metallo-supramolecular gels obtained from the self-assembly of metal ions and multitopic cyclam bis-terpyridine ligand **54**, and these metallo-supramolecular gels were proven to be thixotropic (Figure 24c) [73].

**Figure 25:**
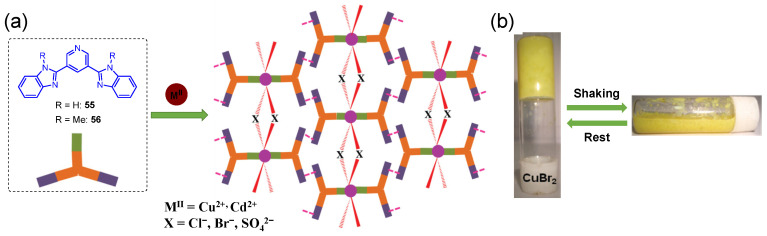
(**a**) Molecular structure of pyridine-3,5-bis(benzimidazole-2-yl) ligands **55** and **56**, and proposed model of the self-aggregation of **55** and metal halides (X). (**b**) Illustration for mechano-responsive nature of **55**-CuBr_2_ gel. Reprinted with permission from Ref. [92]. Copyright (2012), American Chemical Society.

In 2012, Biradha et al. found that pyridine-3,5-bis(benzimidazole-2-yl) ligands **55** [92] and **56** [93] could form stable metallogels in aliphatic alcohols following coordination with transition metal salts (e.g., Cu(II) or Cd(II)). The pyridine N-atom of the ligands was coordinated with the metal ion to form a discrete complex. The intermolecular hydrogen bonding drove the complexes to self-assemble into a cross-linked network for holding the solvent molecules (Figure 25a). The formed metallogel was found to be thixotropic. The yellow gel was changed into a free-flowing liquid after vigorous shaking for a few minutes, and the resulting liquid was restored to a gel after resting (Figure 25b).

**Figure 26:**
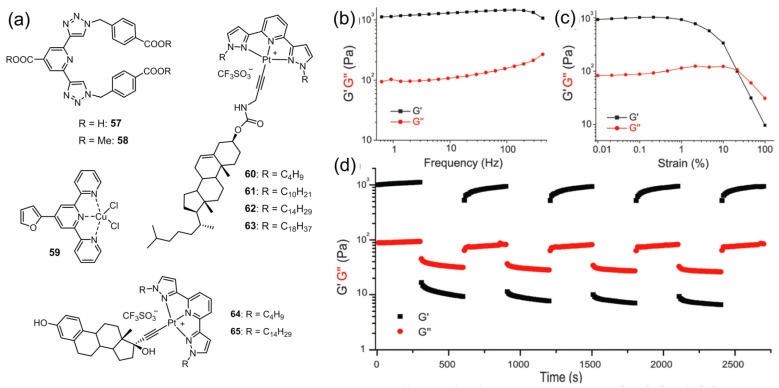
(**a**) Chemical structures of **57**–**65**. Oscillatory rheology measurements of **57**-Eu(III) gel. (**b**) Frequency sweeps at 0.1% strain amplitude of the storage modulus G’ and loss modulus G’’ are shown. (**c**) The corresponding strain dependence at af = 1 Hz. (**d**) Recovery test for **57**-Eu(III) gel with alternating strain amplitudes of 20% and 0.1% at f = 1 Hz. Reprinted with permission from Ref. [94]. Copyright (2015), Royal Society of Chemistry.

Gunnlaugsson et al. designed a series of new 2, 6-bis(1, 2, 3-triazol-4-yl)pyridine (btp) ligands **57, 58** (Figure 26a) [94]. A luminescent supramolecular metallohdydrogel was formed from the ligand and a Eu(III) ion with a 1:3 (Eu:L) stoichiometry ratio. Rheological studies proved that the metallohdyrogel exhibited thixotropic behavior (Figure 26b-c). The storage modulus G′, associated with energy storage, and the loss modulus G″, associated with loss of energy, were monitored as functions of frequency. The value of G′ was more than one order of magnitude greater than that of G″, indicating a stable gel. The G′ and G″ of the gels exhibited slight frequency dependence in the frequency range, demonstrating that the gels had good tolerance to external forces (Figure 26b). As shown in Figure 26c, at low strain values, the value of G′ was larger than that of G″, suggesting an elastic characteristic of the sample. Both moduli remained roughly constant below a critical strain value of 20%, known as the upper limit of the linear viscoelastic regime. Above this value, a sharp decrease in G′ and G″ was observed, representing a partial destroying of the gel. The metallogel system changed from liquid-like (G″ > G′) to solid-like (G′ > G″) behavior almost instantly with a quick recovery of within 50 s of the original values of the moduli in these two regimes; this occurred consistently and over consecutive runs (Figure 26d). Tu et al. found a pincer-type terpyridine Cu(II) complex **59**, which was able to form metallogels in water, glycol and glycerol with a low CGC (Figure 26a) [95]. The weak interactions, including π–stacking, metal−metal interactions and hydrogen-bonding interactions between gelators and guest molecules, were responsible for gel formation. The formed metallohydrogel was found to be thixotropic. A family of cholesterol-/estradiol-appended alkynyl platinum(II) complexes **60**–**65** with tridentate N-donor ligands was synthesized by Yam’s group (Figure 26a) [96]. The complexes with longer N-alkyl chains were able to form stable metallogels in decalin and cyclohexane. Shaking the metallogel using a vibrator broke the gel completely, and the gel state reformed after resting. The authors believed that the mechanical force was sufficient to break the weak noncovalent interactions and would result in a disassembly process. After resting, the broken noncovalent interactions had a strong tendency to reform, which drove the molecules to re-assemble and form a supramolecular network that was responsible for gelation.

**Figure 27:**
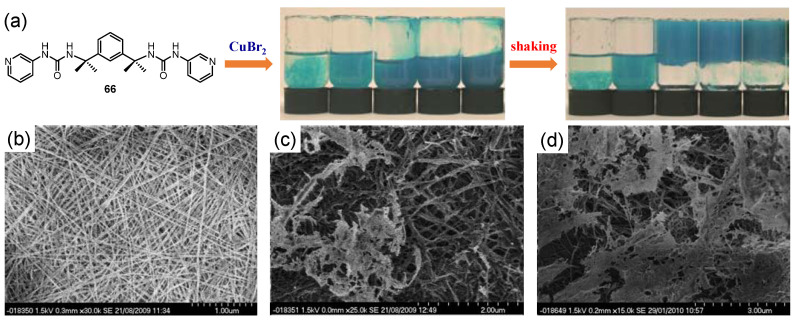
(**a**) CuBr_2_ metallogels of ligand **66** (1 wt%) in MeOH using (from left to right in each picture) 0.1, 0.2, 0.3, 0.4 and 0.5 equivalents of CuBr_2_. The left-hand picture shows thick flowing liquids before shaking, and the right-hand picture shows the same samples after shaking and gelation of the 0.3, 0.4 and 0.5 systems. Cryo-SEM images of a gel sample of **66** in MeOH with 0.3 equivalents of CuBr_2_ (**b**) before shaking, at −100 °C and (**c**) after shaking, at −80 °C, and (**d**) the shaken sample after 1 week of resting, at −70 °C. Reprinted with permission from Ref. [97]. Copyright (2010), Royal Society of Chemistry.

Interestingly, Steed et al. reported an example of shear-induced gelation in a copper(II) metallogel with a pyridyl-urea based ligand **66** [97]. In this metallogel system, rapid shaking of the samples resulted in transformation from a weak gel-like material to a robust gel (Figure 27a), which was different from the above-mentioned thixotropic metallogels. The authors believed that mechanical shaking caused a prominent increase in the number and connectivity of the nodes within the gel network. The effect of mechanical stress on the morphology was studied via cryo-SEM. Many straight, rigid uniform fibers with diameters in the range of 5–8 nm, and with limited interconnecting nodes, were observed before vigorous shaking (Figure 27b), indicating that the fibers could freely slide over each other. After shaking, the gel matrixes were much more fused and interconnected, and the fibers were even less homogeneous, with thick strands ~20 nm in diameter (Figure 27c). The fusion of the individual fibers continued even after leaving the shaken metallogel to stand for several weeks (Figure 27d). Therefore, shaking led to transformation of the system from a bundle of homogeneous and one-dimensional fibers (not extensively interconnected) to a three-dimensional gel network with a high cross-linking density.

**Figure 28:**
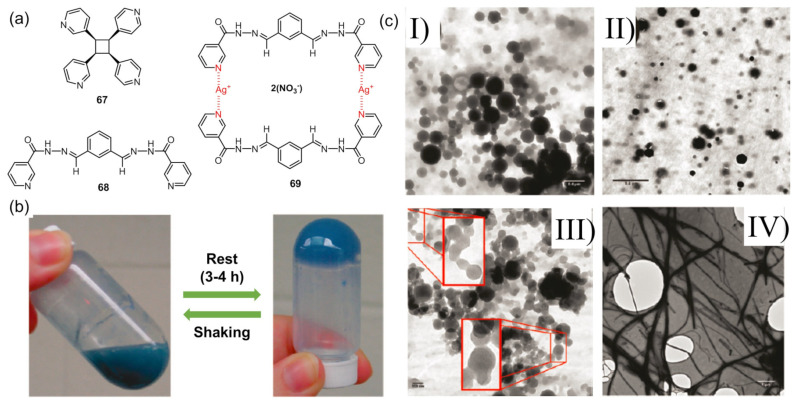
(**a**) Chemical structures of **67**-**69**. (**b**) Metallogels upon combining Cu(BF_4_)_2_•3H_2_O and **67** in water. (**c**) TEM micrographs of the hydrogel **Cu-67**: (I) fresh gel upon standing (>4 h), (II) sol after shaking (5 min), (III) transition of NMOPs of reformed gel into “pearl-necklace” structures (in red) (3-6 weeks), and (IV) nanobundles in aged gels (about a year). Reprinted with permission from Ref. [98]. Copyright (2011), American Chemical Society.

MacGillivray and coworkers reported a series of metallogels from Cu(II) and tetratopic ligand **67** in water and polar solvents including acetonitrile, nitromethane and methanol, with various counter-anions (Figure 28a) [98]. The metallohydrogels composed of nanoscale metal–organic particles (NMOPs) showed thixotropic performance (Figure 28b). The effect of shaking on the morphology of the metallogel were studied via TEM. Representative images of the freshly prepared gel revealed spherical NMOPs with approximately 50–300 nm diameters. The Spherical NMOPs remained the main aggregates in the immediately formed sol, triggered via shaking, and the reformed gel. A morphological transition from NMOPs to nanobundles and a 2D film of the gel were observed after aging for weeks. Even prolonged aging time (~1 year) resulted in aggregated and interwoven networks of nanobundles (Figure 28c). A shear stress loop test was used to quantify the thixotropic behavior of the metallogel. Extrapolation of the straight line portion of the curve to the stress axis was determined to give a yield value of 8.33 Pa. Ligands **68** with two pyridinyl groups were linked via coordination between the silver ion and N-atom of the pyridine to give a macrocyclic basic unit **69** of the self-assembly process [99]. Then, two or more basic units were assembled together via hydrogen bonding to form tetragonal or hexagonal packing modes. The formed metallogel showed a fully reversible thixotropic property, which was further proved by rheological experiments.

**Figure 29:**
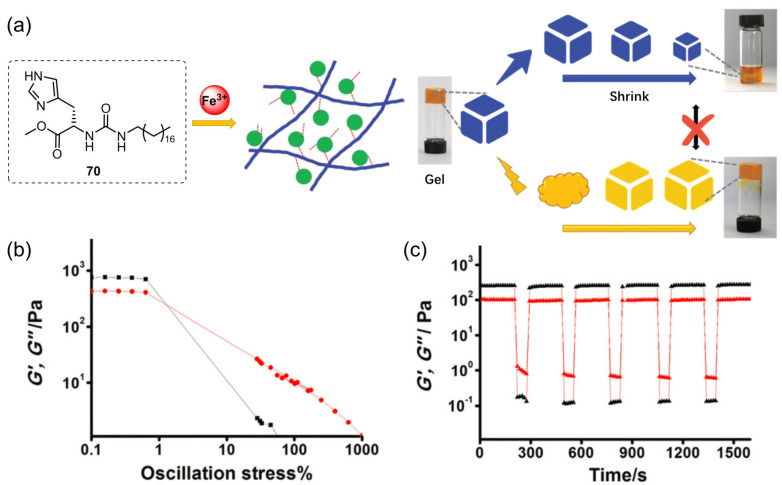
(**a**) Molecular structure of the gelator **70** with metal ions and illustration of the properties of the hydro-metallogel. The ferric gel shrunk without disruption. If the as-prepared gel was shaken immediately, the gel showed thixotropic properties. (**b**) Strain dependence at 6.28 rad s^−1^; (**c**) continuous-step measurement of the ferric hydrogels formed in a mixture of **70**/Fe^3+^ solution (12 mM **70**; 24 mM Fe^3+^) with alternation of the strain amplitude between 0.5% and 10% (black: G’, red: G’’). Reprinted with permission from Ref. [100]. Copyright (2016), Royal Society of Chemistry.

Liu’s group designed a new metallohydrogelator consisting of an amphiphilic L-histidine derivative **70** and ferric ions (Figure 29a) [100]. The as-formed metallohydrogel was changed into a sol if the gel was shaken or shear stress was applied. The collapsed sol was transformed into a stable gel state upon resting for a few minutes. Rheological experiments were further used to study the thixotropic properties of the metallohydrogel. The strain amplitude sweep results indicated that both G′ and G″ decreased rapidly at critical strain. Once the value of G′ was lower than that of G″, a gel–sol phase transition occurred (Figure 29b). However, the value of G′ increased sharply and was larger than that of G″ when the amplitude oscillation was canceled, which indicated a sol–gel phase transition. This thixotropic behavior could be repeatedly carried out many times (Figure 29c). The authors believed the thixotropy process of the metallohydrogel under shear stress was due to the flexibility of the coordination of the ligand **70** with the metal ions. Metal–ligand interactions slackened upon shear stress, and reformed when shear force disappeared. Interestingly, with increased resting at room temperature, the metallogel underwent shrinkage, and ~90% of the ferric water was expelled from the gel matrix (Figure 29).

### 2.3. Multi-Stimuli-Responsive Metallogels

Compared with metallogels that respond to a single stimulus, metallogel systems that produce an intelligent response to multiple external stimuli (more than two kinds of stimuli) have drawn increasing interests due to their excellent multiple stimuli-triggered dynamic and reversible property changes. In this section, multiple stimuli-responsive metallogels are classified into discrete metal complexes-, metallopolymers and host–guest chemistry-based multi-stimuli-responsive metallogels.

#### 2.3.1. Discrete Metal Complex-Based Multi-Stimuli-Responsive Metallogels

**Figure 30:**
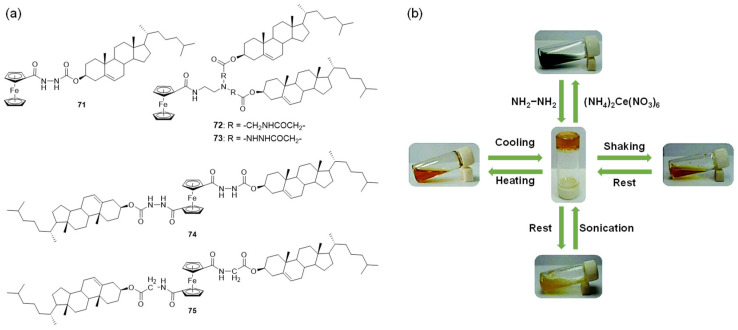
(**a**) Cholesterol-appended ferrocene gelators **71**–**75**. (**b**) Reversible sol–gel phase transition of the gel of **71**/cyclohexane, triggered by chemical redox reaction, shear stress, sonication and temperature. Reprinted with permission from Ref. [101]. Copyright (2008), John Wiley and Sons.

A variety of discrete metal complexes with precise molecular structures have been found to form stable metallogels. They can respond to multiple environmental stimuli, including anions, neutral chemicals, light, sonication, mechanical stress, etc. An early example of a multiple stimuli stimuli-responsive metallogel was reported by Fang’s group [101]. The metallogelators **71**–**75** contained redox-active ferrocenyl groups and cholestery residue linked via amide linkage (Figure 30a) [101,102,103]. The metallogelator **71** was able to form a stable yellow gel in cyclohexane with CGC as low as 0.09 wt% elevating it to the category of supergelator [101]. As expected, an immediate sol–gel phase transition was induced by adding (NH_4_)_2_Ce(NO_3_)_6_. In contrast, stirring or shaking the solution for a few seconds resulted in gelation, again, when hydrazine was added. The stable yellow gel was converted to a yellow sol by shaking the gel vigorously, and the gel state was recovered after standing for minutes. In addition, the formed metallogel **71** showed a reversible gel–sol phase transition in response to sonication and temperature (Figure 30b). Moreover, the metallogelator **72** with two cholesterol units and one Fc group designed by the same group was able to gel various organic solvents [102]. The gel of **72**/n-decane showed thermal, mechanical, acoustic and chemical stimulus-responsive reversible sol–gel state transition properties. The metallogels formed from the complexes 74-75 responded to multiple stimuli, including temperature, oxidants and shear stress [103].

**Figure 31:**
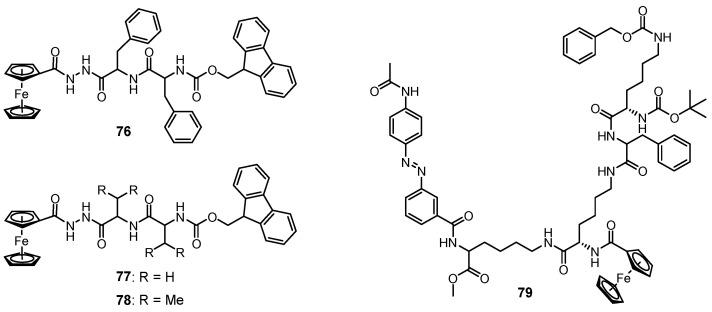
Chemical structures of **76**–**79**.

Zhang et al. found that ferrocenoyl phenylalanine monomer **31** aggregated in water to form a metallohydrogel, and the formed metallohydrogel showed a phase change in response to a series of disparate stimuli such as oxidation–reduction reactions, guest–host interactions and pH changes [61]. In 2014, Yu et al. designed a class of ferrocene-peptide complexes **76–78** (Figure 31) [104]. Some of them were able to gel various organic solvents and mixed solvents. The metallogel **76** in isopropanol–water (*v/v*, 1/1) was found to show a reversible gel–sol phase transition under different stimuli. (NH_4_)_2_Ce(NO_3_)_6_/hydrazine hydrate as an oxidant/redox pair was able to successfully trigger a gel–sol state transition due to the alternate oxidation and reduction of the ferrocene moiety. In addition, the metallogel responded to guest molecules such as β-CD, accompanied with a gel–sol transition, as a result of host–guest interactions between β-CD and ferrocene. Kratz et al. designed a ferrocene–dipeptide conjugate **79** with a photochromic azobenzene group (Figure 31) and found that these discrete metallogelators could form metallogels in various organic solvents [105]. The formed metallogels responded to various external signals, including ultrasound irradiation, temperature, redox and mechanical stress, showing a reversible gel–sol phase transition. Importantly, the resulting gel was changed into a dark-orange solution after irradiation with UV light. The resulting solution was reformed to a gel state after blue light irradiation or sonication treatment. The isomerization of the azobenzene groups between trans- and cis-configuration was responsible for the photo-responsiveness.

**Figure 32:**
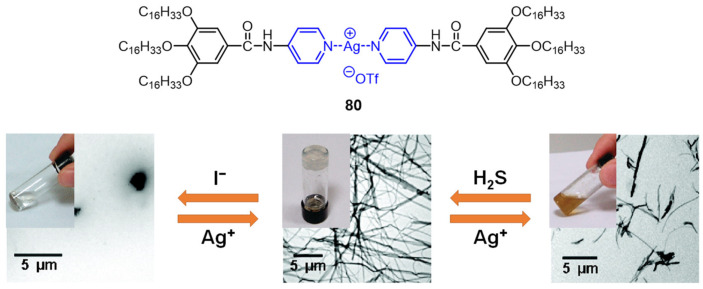
Photographs of the response property of the Ag-coordinated complex gel **80** toward potassium iodide and H_2_S gas, and the corresponding TEM images. Reprinted with permission from Ref. [106]. Copyright (2007), American Chemical Society.

A silver-coordinated complex **80** consisting of a silver(I) ion and two organic components with a pyridyl head group was synthesized by Wu et al. [106]. Hydrogen bonding and the Van der Waals interaction played an important role in the gel formation. The formed metallogel showed a rapid response to wide range of chemical stimulation, such as anions including I^−^, Br^−^, and Cl^−^, neutral chemicals such as H_2_S and NH_3_, and pH value changes (Figure 32).

**Figure 33:**
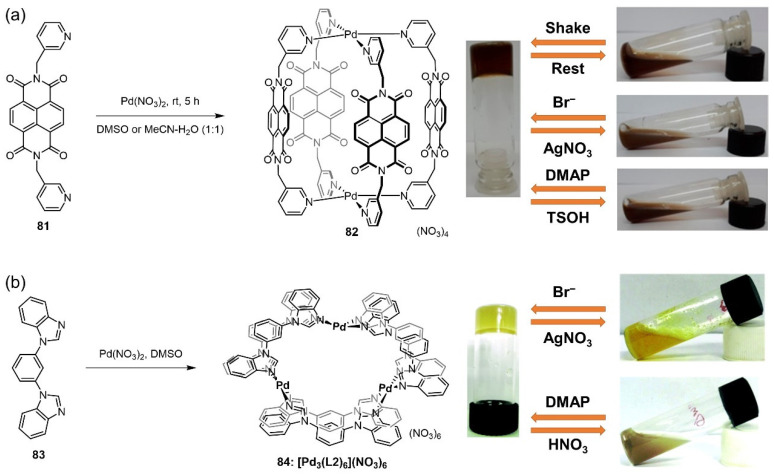
(**a**) Chemical structures of the metallic cage **82** and photographs of reversible stimuli-responsive phase transition of the gel **82**. Reprinted with permission from Ref. [107]. Copyright (2018), American Chemical Society. (**b**) Chemical structures of the trinuclear ring **84** and photographs of reversible stimuli-responsive phase transition of the gel **84**. Reprinted with permission from Ref. [108]. Copyright (2015), Royal Society of Chemistry.

A self-assembled binuclear cage **82** of the Pd_2_L_4_ formulation consisting of Pd(II) ions and naphthalenediimide moieties **81** was found to gel DMSO and an acetonitrile–water mixture [107]. The resulting metallogel displayed a thixotropic nature and showed a gel–sol phase transition after the alternating addition of TBABr/AgNO_3_ and DMAP/TsOH reagent pairs due to the disassembly and reassembly of the complex through ligand-exchange reactions (Figure 33a). In 2015, a variety of coordination rings **84** with an M_3_L_6_ composition were fabricated through the combination of Pd(NO_3_)_2_ with a benzimidazolyl-appended bidentate non-chelating ligand **83** by Chand’s group [108]. A solution of [Pd_3_L_6_] in DMSO formed an opaque supramolecular metallogel after a few minutes of stirring. These metallogels showed a reversible stimuli-responsive gel–sol phase transition caused by the disassembly and re-assembly of M_3_L_6_ coordination rings based on the dynamic nature of the metal–ligand coordination. For example, the DMAP-HNO_3_ and ethylenediamine-Pd(NO_3_)_2_ reagent pairs successfully triggered a gel–sol phase transition in these metallogels (Figure 33b).

**Figure 34:**
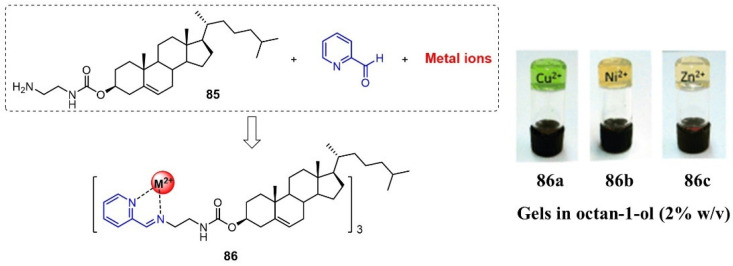
Preparation of gelators **86a**–**c** via subcomponent self-assembly. Reprinted with permission from Ref. [109]. Copyright (2013), John Wiley and Sons.

Bunzen et al. prepared a new class of metallogels via subcomponent self-assembly through a combination of pyridine carboxaldehyde **85**, steroidal amine and different metal ions (i.e., Cu(II), Ni(II) and Zn(II) ions) [109]. The formed complex **86** was able to form stable metallogels in alcohols, aromatic solvents and CCl_4_ (Figure 34). These formed metallogels were found to be responsive toward a variety of chemicals (i.e., EDTA) and physical stimuli.

**Figure 35:**
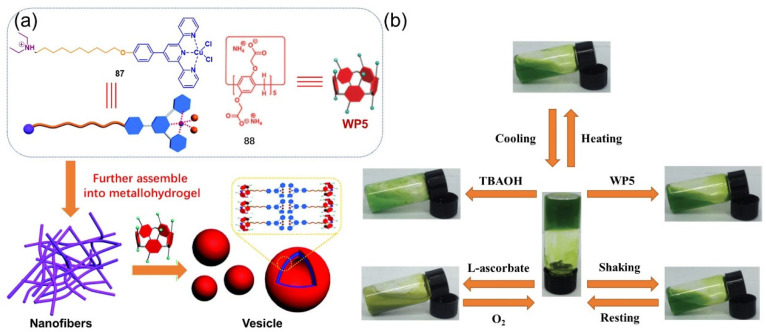
(**a**) Chemical structures of ligands **87** and anionic water-soluble pillar[5]arene WP5 **88**, and cartoon representations of the gelation process and WP5-induced morphology transformation. (**b**) Photographs of the gel–sol transitions of the metallohydrogel triggered by different stimuli. Reprinted with permission from Ref. [110]. Copyright (2015), Royal Society of Chemistry.

In 2015, Yao et al. reported that a terpyridine-based low-molecular-weight ligand **87** and a metallohydrogel were formed after coupling with divalent cooper ions under acidic conditions [110]. The formed metallohydrogel was found to be responsive toward multiple stimuli (i.e., temperature, shear stress, L-ascorbate, TBAOH and pillararene) (Figure 35b). A reversible gel–sol state transition upon alternating shaking and resting suggested a thixotropic nature. Adding excessive sodium L-ascorbate into the hydrogel resulted in the formation of precipitates because of the reduction of Cu(II) to Cu(I) ions. The metallogel was reconstructed by oxidizing the precipitates with oxygen. In addition, a few drops of TBAOH solution resulted in the formed metallohydrogel being gradually disrupted because of the formed Cu(OH)_2_ precipitates. A water-soluble pillararene WP5 **88** was also found to trigger a gel–sol phase transition with the assistance of host–guest interactions. The metallogel collapsed into a sol after the addition of pillar[5]arene (WP5). A morphology change from nanofibers in the gel state to vesicles in the solution was observed during the response process (Figure 35a). The formed host–guest complexes acted as supra-amphiphiles and self-assembled into bilayer vesicles.

**Figure 36:**
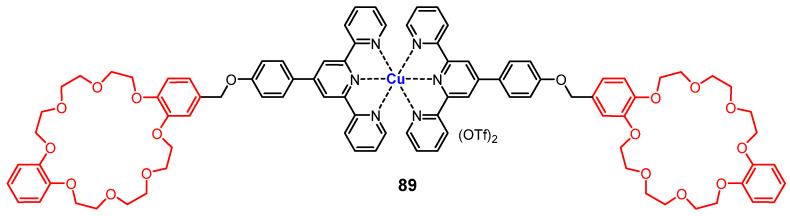
Chemical structures of supra-amphiphile complex **89**.

Recently, Du and coworkers reported a new supra-amphiphilic metallogelator **89** (Figure 36), with a dibenzo-24-crown-8 as a hydrophilic part and a hydrophobic complex part containing copper ions and terpyridine groups [111]. The metallogelator formed light-green metallogels, and the formed supramolecular gels showed quadruple stimuli-responsiveness performance under stimulation with heat, ultrasound and chemicals. The metallogel ruptured and finally turned into a green solution upon exposure to TFA vapor. The gel–sol phase transition was probably caused by the breaking of metal–ligand coordination due to the protonation of pyridine. The gel was restored after being exposed to TEA vapor. A reversible gel–sol phase transition was also triggered by alternately adding K^+^ and benzo-18-crown-6 through competitive host–guest interactions.

**Figure 37:**
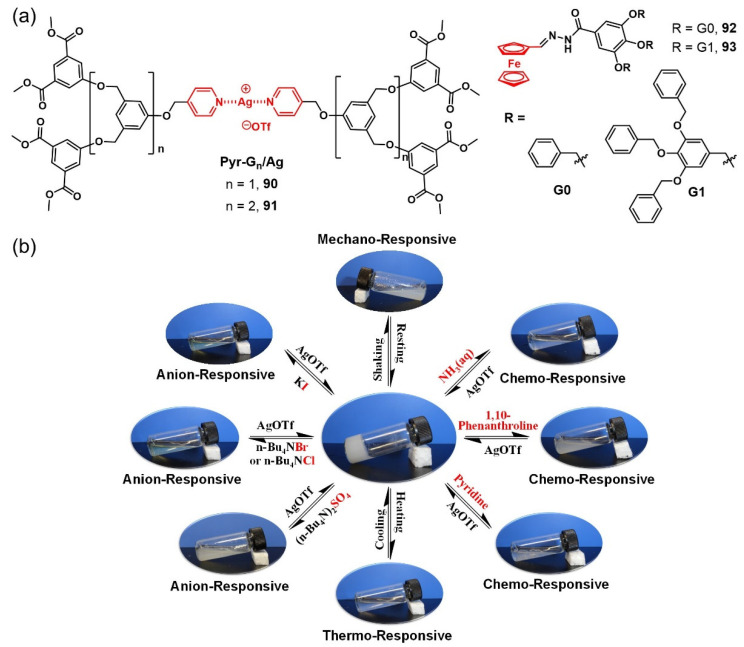
(**a**) Chemical structures of dendritic metallogelators **90**-**93**. (**b**) Reversible sol–gel phase transition of the metallogel triggered by shear stress, temperature and the presence of anions and chemicals. Reprinted with permission from Ref. [114]. Copyright (2014), John Wiley and Sons.

Dendrimers and dendrons are highly branched macromolecules with well-defined molecular structures and have been widely used as building blocks in the self-assembly of supramolecular gel-phase materials [112,113]. Their unique dendritic architecture renders them capable of being modified with metal ions into different positions, such as the dendritic core, the tether termini, the dendritic branches or the branching points, to develop multiple stimuli-responsive metallogel systems. In 2014, Fan’s group designed and synthesized a new class of poly(aryl ether) dendritic ligand with pyridine functionality at the focal point and corresponding Ag(I) complexes **90**–**91** (Figure 37a) [114]. The organometallic dendrimers showed better gelation ability towards various organic solvents with much lower CGCs. The multiple noncovalent interactions, including metal–dendritic ligand coordination, solvophobic interactions and π–π stacking, cooperatively promoted the gelation process. These dendritic metallogels were found to be multiple stimuli-responsive supramolecular gels (Figure 37b). They showed a quick response to some anions, such as the chloride ion, bromonium ion, iodine ion and sulfate ion, showing a gel–sol transformation. Excess silver trifluoromethanesulfonate (AgOTf) triggered a sol–gel phase transition. Additionally, these metallogels were also able to respond to neutral chemicals such as pyridine, ammonia and 1,10-phenanthroline, but the response process was not reversible. The thixotropic property was investigated in detail using rheological techniques, and the thixotropic process could be repeated many times. These dendritic metallogels were further used as templates for the in situ formation and stabilization of silver nanoparticles without the use of any chemical reducing/stabilizing agents. Prasad et al. reported another multi-stimuli-responsive organometallic gel based on ferrocene-linked poly(aryl ether) dendrons **92–93** (Figure 37a) [115]. The dendritic metallogelator **93** formed robust stable metallogels in both polar and non-polar solvent/solvent mixtures. This dendritic metallogel was thermally reversible, and redox- and chemical-responsive. The gel was changed into a sol and finally became a dark-green solution after stimulation with anodic potential or an oxidant such as [Ce(NH_4_)_2_(NO_3_)_6_]. The resulting solution was transformed into a stable gel after a short period of sonication irradiation. The reversible gel–sol phase transition was attributed to increased repulsion between neighboring ferrocenyl ions during the oxidation process, resulting in a breakdown in intermolecular H-bonding and π–π stacking interactions. These dendritic metallogels also showed a specific response to Pb (II) ions triggering the gel–sol transition, suggesting that they could be an easy detection kit for lead ions, with a detection limit close to the ppb level.

#### 2.3.2. Metallopolymer-Based Multi-Stimuli-Responsive Metallogels

**Figure 38:**
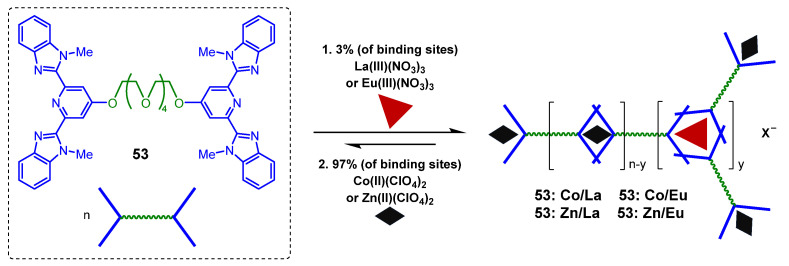
Schematic representation of the formation of a metallo-supramolecular gel-like material using a combination of lanthanoid and transition metal ions mixed with a monomer **53**.

Metallopolymers obtained from the self-assembly of metal ions and polytopic bridging ligands are promising for the development of metallogels with external multiple stimuli-responsiveness [33]. Rowan et al. designed and synthesized a series of metallo-supramolecular polymers with stimuli-responsive behavior through a combination of a polytopic ligand **53** containing a bis(2,6-bis(1′-methylbenzimidazolyl)-4-hydroxypyridine) moiety and certain proportions of a transition metal ion Zn(II) and a lanthanide ion La (III) (Figure 38) [90,116]. The metallo-supramolecular gels were spontaneously formed after adding lanthanoid(III) nitrate (3 mol %), and then, the transition metal ion perchlorate (97 mol %) to a solution of the polytopic ligand in organic solvents. The resulting metallogel showed thermo-, chemo- and mechano-responses. A gel–sol phase transition was observed at high temperatures due to a thermally broken La (III)/ligand coordination. These metallogels also exhibited thixotropic properties. A free-flowing liquid was obtained after vigorous shaking. The gel state was restored upon standing for a few seconds. The metallopolymer was observed to self-assemble into globular colloidal particles with spherulitic structures during the gelation process. Its fragile nature and sensitivity to mechanical perturbation were responsible for its thixotropic property. In addition, the presence of lanthanide(III) ions reduced the crystallinity of the colloidal particles and increased their mechano-responsiveness due to the different coordination abilities of the lanthanide(III) and zinc(II), which changed the nature of the self-assembly in the gel state. Recently, a film with multi-responsive shape-memory properties was prepared using covalently cross-linked metallo-supramolecular polymers [117]. These films consisted of a soft poly(butadiene) phase and a hard metal–ligand phase. The stimuli, such as temperature, light and chemicals, which could soften the hard phase, were used to tune the shape-memory properties of these films.

**Figure 39:**
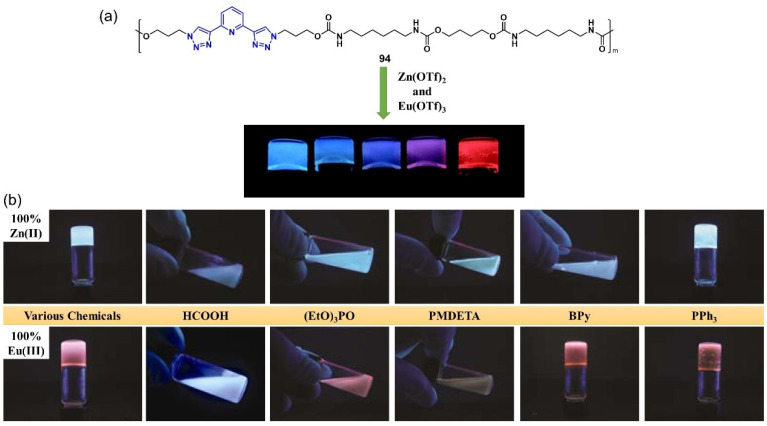
(**a**) Picture of gels (20 mg mL^−1^ in chloroform) derived from polymer **54** with varying amounts of Zn(II) and Eu(III) (from left to right, Zn/Eu: 100/0, 75/25, 50/50, 25/75 and 0/100) under 365 nm UV light, demonstrating the effects of different metal combinations on the photoluminescence color. (**b**) Chemo-responsiveness of gels to various chemicals. Reprinted with permission from Ref. [118]. Copyright (2012), Royal Society of Chemistry.

A metallo-supramolecular gel was prepared by combining a ligand macromolecule **94** containing a 2,6-bis(1,2,3-triazol-4-yl)pyridine (BTP) group in the polymer backbone with transition metal ions (Zn^2+^) and/or lanthanide ions (Eu^3+^) (Figure 39a) [118]. The gelation process and the gel properties could be finely controlled by a selection of metal ions and their combinations, solvent, concentration, etc. The formed metallogel was found to respond to temperature and chemicals (e.g., bipyridine and the nerve gas agent mimic triethyl phosphate) (Figure 39b). Interestingly, the metallogels showed repeatable autonomic healing ability.

**Figure 40:**
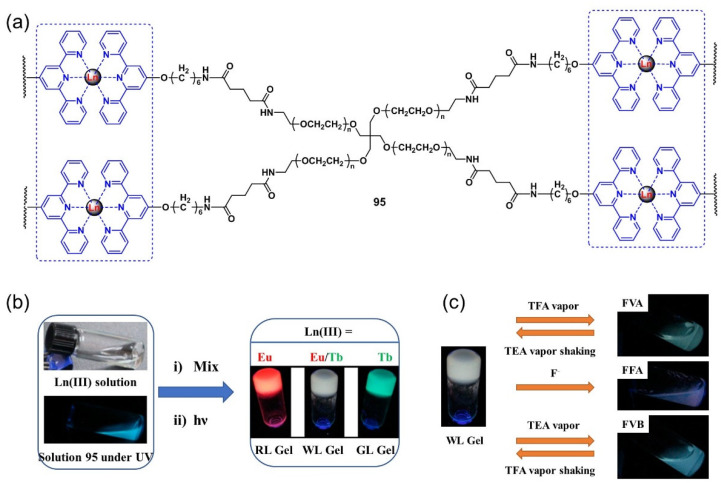
(**a**) Chemical structure of polymer **95** cross-linked via Ln−Terpy coordination. (**b**) Schematic representation of Ln(III) coordination-based luminescent metallogels under UV light (λ_ex_ = 365 nm, 3.5 wt % polymer **95**, DMF/CH_3_CN = 1:1, *v*/*v*). (**c**) Stimuli-responsive emission color changes and phase transitions of WL Gel. Reprinted with permission from Ref. [119]. Copyright (2015), American Chemical Society.

Holten-Andersen et al. designed a light-emitting metallogel formed of a coordination of terpyridyl-end-capped four-armed poly(ethylene glycol) polymer ligands **95** with lanthanide metal ions (Figure 40a,b) [119]. The formed metallogel underwent a reversible gel–sol phase transition and a color change in response to stimuli including temperature, mechanical stress, chemicals, etc. A gel–sol phase transition correlated with color changes was observed after exposure to trifluoroacetic acid (TFA). The gel state and color were restored upon treatment with triethylamine (TEA). The addition of an F^−^ anion also induced a gel–sol phase transition and a color change due to the competitive interactions between lanthanide metal ions and F^−^ anions (Figure 40c). However, this responsive process was irreversible because of the formation of LnF_3_ precipitates. The corresponding coatings and films formed from the above metallogelator also showed multi-stimuli-responsive properties.

**Figure 41:**
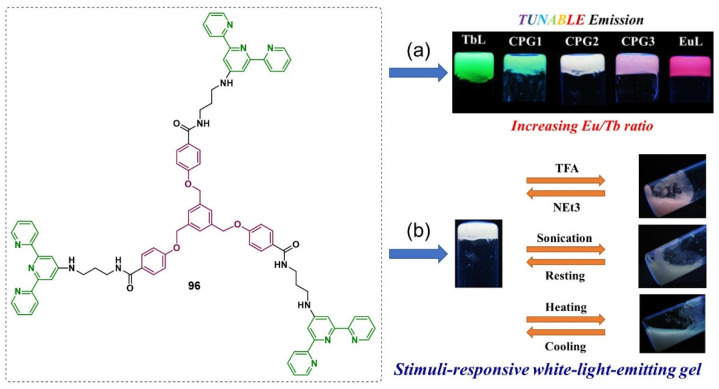
(**a**) Coordination of **96** with Tb^III^/Eu^III^ (in different ratios) resulted in CPGs with tunable emission properties, including white-light emission. (**b**) The white-light-emitting CPG showed multi-stimuli-responsive behaviors. Reprinted with permission from Ref. [120]. Copyright (2017), American Chemical Society.

Maji et al. designed an amphiphilic, tripodal low-molecular weight ligand **96** consisting of a 4, 4′, 4-[1,3,5-phenyl-tri(methoxy)]-tris-benzene core and 2,2′:6′,2′′-terpyridyl termini [120]. The coordination of the ligand with different metal ions (i.e., Tb(III)/Eu(III)) in different ratios provided a series of metallogels with tunable emission properties, including white-light emission (Figure 41a). The white-light-emitting metallogel showed responsiveness to physical (i.e., temperature and sonication) and chemical stimuli. When it was irradiated with sonication for a few minutes, the metallogel was gradually disturbed. This state transition was attributed to the sonication-induced cleavage of Ln-N_tpy_ coordination bonds, which affected the stability of the gel network. A reversible gel–sol state transition was observed when the white-light-emitting metallogel was alternately treated with the vapor of trifluoroacetic acid (TFA) and triethylamine(NEt_3_) (Figure 41b). The protonated terpyridine (tpyH^+^) had a lower affinity for Ln(III) ions due to increased electrostatic repulsion. Thus, the metallogel was disturbed and showed a gel–sol phase transition.

**Figure 42:**
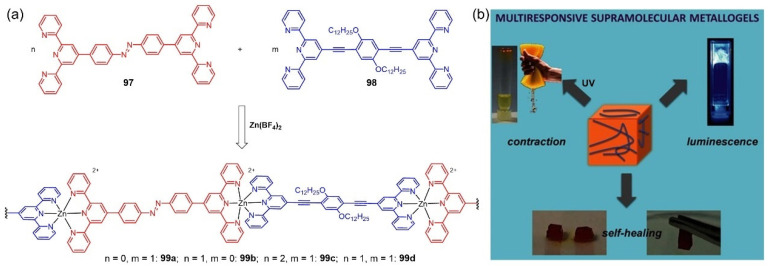
(**a**) The structures of metallo-copolymers **99**. (**b**) Schematic representation of the smart properties, including light-powered actuation, photoluminescence and self-healing. Reprinted with permission from Ref. [121]. Copyright (2016), John Wiley and Sons.

Mauro et al. first synthesized two different terpyridyl-based ligands **97** and **98** [121]. The ligand **97** consisted of two terpyridyl groups and an azobenzene linkage, and the other ligand **98** contained ditopic terpyridyl bridged by π-conjugated small phenylene ethynylene. When the two different ligands and Zn(II) ions were mixed in a specific ratio, a series of luminescent metallogels were formed in a DMF/ethanol (1:20, *v/v*) mixed solvent (Figure 42a). The resulting metallogels displayed light-triggered mechanical actuation and luminescent properties (Figure 42b). The as-prepared metallogels had good self-healing properties owing to the presence of dynamic and reversible metal–ligand bonds. The metallogel acted as a light-triggered soft actuator due to the trans-to-cis photoisomerization process of the azobenzene under UV light irradiation. This metallogel could be made into highly ordered patterns, and the reversible pattern switching could be achieved using UV-visible light irradiation.

**Figure 43:**
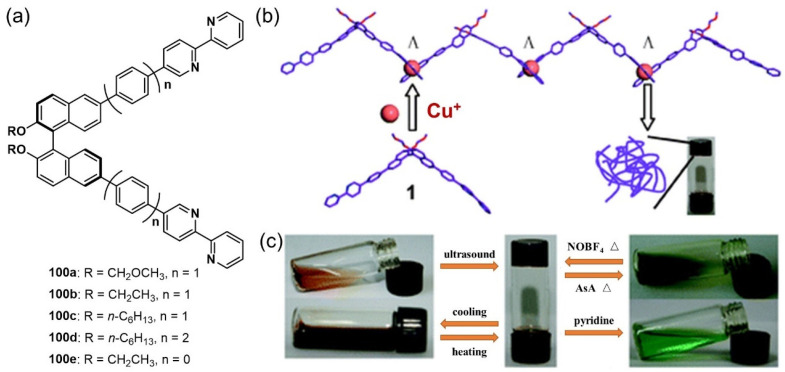
(**a**) The structures of ligands **100**. (**b**) A schematic representation of the possible self-assembly processes of the coordination polymer gel Cu(I)-**100**. (**c**) Phase transition behavior of the gel Cu(I)-**100** triggered by sonication, temperature, chemical redox reaction and solvent. Reprinted with permission from Ref. [122]. Copyright (2010), Royal Society of Chemistry.

Pyridine, bipyridine and their derivatives are some of the most extensively used ligands for forming complex metallic centers. They are widely used as building blocks to develop stimuli-responsive metallogel systems. In 2010, a chiral binaphthylbisbipyridine ligand **100** (Figure 43a) was reported by Bian et al. [122]. A complexation of Cu(I) ions and the ligands **100a** triggered a gelation process in a CH_3_CN–CH_2_Cl_2_ (*v/v*, 1/1) solution (Figure 43b). The metallogel was able to respond to various stimuli, including temperature, sound, pyridines and redox (Figure 43c). A reversible gel–sol phase transition was observed under alternate stimulation with oxidant NOBF_4_ and reductant ascorbic acid. The dark-red gel was turned into a light-green solution when pyridine was added. The metallogel was also used as a self-supported stable supramolecular catalyst for the click reaction.

**Figure 44:**
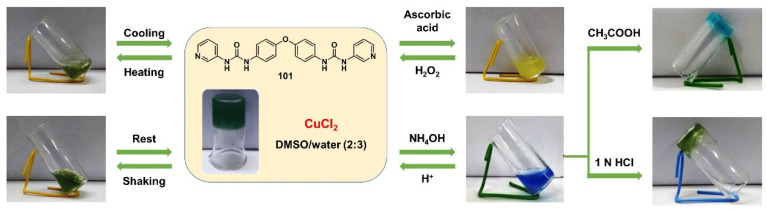
Molecular structure of ligand **101** and the stimuli-responsive behavior of **Cu-101** gels. Reprinted with permission from Ref. [123]. Copyright (2018), John Wiley and Sons.

In 2018, a bis-pyridyl ligand **101** with a diphenyl ether backbone was synthesized and further assembled into a crystalline coordination polymeric network with metal ions [123]. The formed metallogel from the ligand with copper ions showed intelligent sensitivity to various external stimuli, including temperature, shear stress, redox and neutral chemicals (Figure 44). A reversible gel–sol transition was achieved via alternating treatment with a reducing agent, such as ascorbic acid, and oxidant H_2_O_2_, because of the switchable redox state change between the Cu(II) and Cu(I). This reversible process resulted in disruption and reconstruction of the network structure. Chelating ligands such as ammonia induced the green metallogel to change into a deep-blue solution due to a competitive coordination effect. Acid further triggered the sol to be restored to a gel state.

**Figure 45:**
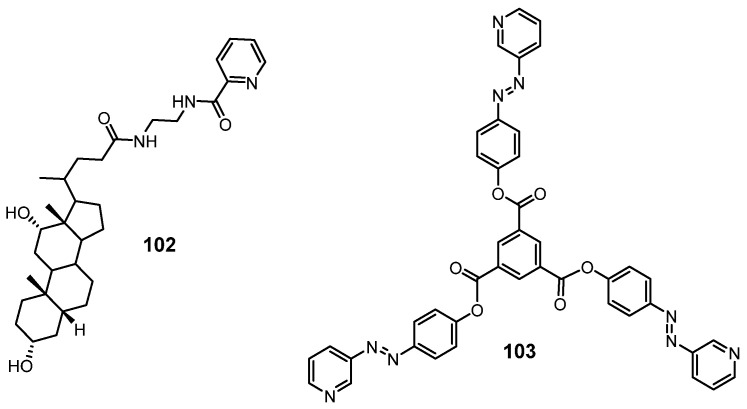
Molecular structure of ligands **102** and **103**.

A picolinic acid-conjugated bile acid derivative **102** (Figure 45) was found to form metallogels in mixed solvents composed of 30–50% of organic solvent in water after coupling with Cu(II) ions [124]. The prepared metallogel could respond to physical stimuli (i.e., sonication and shaking). A gel–sol state transition was triggered by a variety of chemical species, including EDTA, Et_3_N, Pyridine and NH_4_OH. These metallogels also showed reversible redox responsiveness. Li et al. found that the coordination of a *C*_3_ symmetric azopyridine ligand **103** (Figure 45) and Ag(I) ions provided a coordination polymer and trans-coordination polymers that could form metallogels in DMF at low CGCs [125]. Gel–sol state transformation was observed in response to a variety of external stimuli, including temperature, mechanical shearing, light and chemicals (i.e., NH_3_, H_2_S).

**Figure 46:**
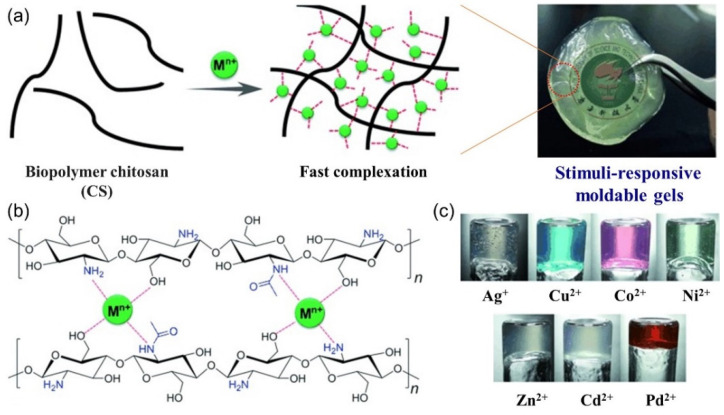
(**a**) Schematic representation of polymer-network hydrogels cross-linked via ultrafast complexation of metal ions and chitosan chains in water. (**b**) Chemical structures of chitosan and their interwoven networks are driven by the complexation between metal ions and OH and NH_2_ groups in the chitosan chains. (**c**) The complexation in (**b**) leads to the formation of hydrogels. Reprinted with permission from Ref. [72]. Copyright (2015), John Wiley and Sons.

However, there are rare examples of the use of native biopolymers for the design of multiple stimuli-responsive metallogels. In 2015, Zhang et al. first reported a metallohydrogel formed via supramolecular complexation of a native biopolymer, chitosan (CS), and transition metal ions, including Ag(Ⅰ), Cu(Ⅱ), Co(Ⅱ), Ni(Ⅱ), Zn(Ⅱ), Cd(Ⅱ) and Pd(Ⅱ) [72]. The gelation process was ultrafast. A variety of transparent and stable metal-hydrogels were obtained within seconds due to the facile coordination of metal ions with amino and hydroxyl groups in the CS chain (Figure 46a,b). The formed metallohydrogel (Figure 46c) was sensitive to a variety of chemical stimuli, including cations (e.g., Cu(Ⅱ)), anions (e.g., S^2−^, Cl^−^, Br^−^, I^−^ and SO_4_^2−^), neutral chemicals (e.g., phytic acids, H_2_O_2_, oxalate, ammonia, etc.) and a pH value change, exhibiting a gel–sol phase transition. This soft matter had remarkable moldability to form shape-persistent, free-standing objects via a fast in situ gelation procedure. Recently, a new self-healing, injectable and adhesive metallohydrogel with multiple-responsiveness was reported through the supramolecular complexation of chondroitin sulfate and Fe(Ⅲ) ions [126]. The hydrogels responded to multiple external stimuli such as ions, neutral chemicals, redox and pH value, accompanied by a gel–sol phase transition. Moreover, this metallohydrogel had strong tissue adhesion that was superior to that of commercial clinical fibrin glue. The stimuli-responsive properties offered a quick and easy approach to removing the adhesive hydrogel from wounds without external force. These adhesive hydrogels have potential for application in next-generation tissue adhesives.

#### 2.3.3. Host–Guest Chemistry-Based Multi-Stimuli-Responsive Metallogels

**Figure 47:**
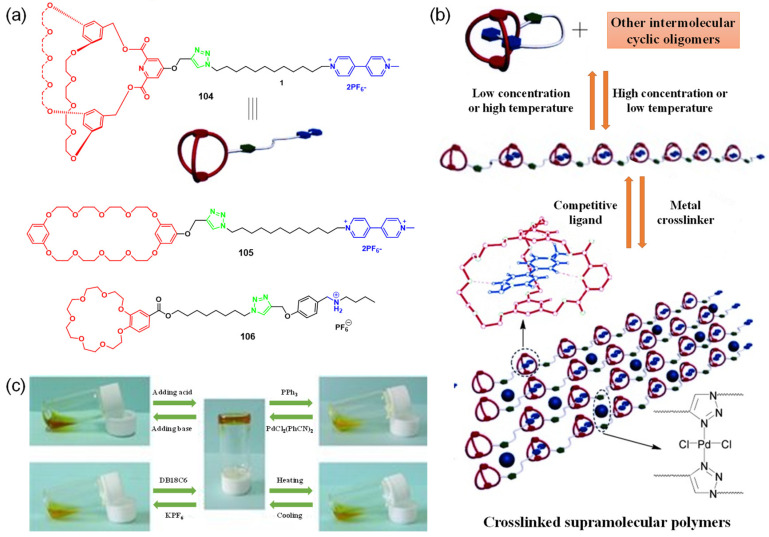
(**a**) Molecular structures of heteroditopic monomers **104**-**106**. (**b**) Schematic representation of controlling the topology and rheology of the supramolecular polymer prepared from heteroditopic monomer **104**. (**c**) The reversible gel–sol transitions of the supramolecular polymer network gel (50 mm of monomer **104**) triggered by four different stimuli (pH-, thermo-, cation- and metallo-induced). Reprinted with permission from Refs. [127,128]. Copyright (2010, 2012), John Wiley and Sons.

A pair of orthogonal noncovalent interactions, including metal–ligand coordination and host–guest interaction, has shown superiority in gluing discrete building blocks together to produce multiple stimuli-responsive metallogels with intriguing performance. In 2010, Huang’s group synthesized two heteroditopic ligands **104**-**105** consisting of a BMP32C10-based host and paraquat guest units linked by a 1,2,3-triazole group (Figure 47a) [127]. Linear supramolecular polymers were found to be formed via host–guest recognition by choosing the proper temperature, ligand concentration and association constants. A transition from liner to cross-linked supramolecular polymers was observed after the binding of 1, 2, 3-triazole in the liner supramolecular polymer with palladium(II) ions (Figure 47b). Dissolution of the supramolecular polymer networks occurred after the addition of a competitive ligand such as PPh_3_ due to the competitive coordination effect. The stimulus-responsiveness was visually confirmed by the formation of a white precipitate. A reversible transition between linear and cross-linked supramolecular polymers was triggered by the successive addition of a metal cross-linker [PdCl_2_(PhCN)_2_] and a competitive ligand PPh_3_. Subsequently, a heteroditopic ligand **106** consisting of a benzo-21-crown-7 (B21C7) host and its complementary guest dialkylammonium salt, linked by a 1,2,3-triazole linkage, was synthesized and characterized (Figure 47a) [128]. A linear supramolecular polymer was first formed in acetonitrile with the assistance of host–guest interactions. Then, the cross-linker [PdCl_2_(PhCN)_2_] was added to trigger a cross-linked supramolecular polymer network by forming a disubstituted palladium(II) complex between the Pd(Ⅱ) ion and 1, 2, 3-triazole moieties, resulting in a supramolecular metallogel. The metallogel showed an interesting reversible gel–sol transition in response to quadruple distinct stimuli (pH-, thermo-, cation-, and metallo-induced) (Figure 47c). Heating–cooling treatment induced a gel–sol phase transition. Triethylamine (Et_3_N) and trifluoroacetic acid (TFA), as the operating acid/base pair, were able to effectively tune the complexation mode of benzo-21-crown-7 (B21C7) and the dialkylammonium salt because of the reversible protonation of the dialkylammonium salt, resulting in a reversible gel–sol transition. The addition of K(Ⅰ)/dibenzo-18-crown-6 (DB18C6) also triggered a gel–sol phase transition. The self-assembled metallogel disassembled when K(Ⅰ) was added. This is because the B21C7 host formed a more stable 1:1 complex with K(Ⅰ), causing the collapse of the gel. The gel was restored after the addition of enough DB18C6 due to the strong affinity of K^+^ and DB18C6, facilitating the reformation of the B21C7/dialkylammonium salt complex.

**Figure 48:**
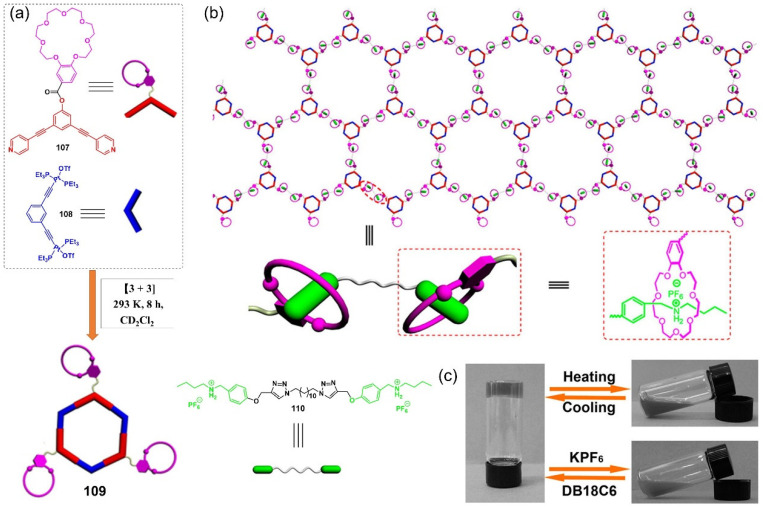
(**a**) Self-assembly of B21C7-functionalized discrete metallacyclic hexagon **109** and (**b**) cartoon representation of the formation of a cross-linked 3D supramolecular polymeric network from self-assembly of hexagon **109** and bis-ammonium salt **110**. (**c**) Reversible gel−sol transitions of supramolecular polymer gels. Reprinted with permission from Ref. [129]. Copyright (2014), American Chemical Society.

In 2014, Huang and Peter J. Stang et al. designed a highly directional dipyridyl ligand **107** containing a B21C7, which was able to form a hexagonal metallacycle with a complementary organoplatinum acceptor **108** via coordination-driven self-assembly (Figure 48a) [129]. A supramolecular cross-linked polymer was formed after the addition of a bis-ammonium salt because of the formation of [2] pseudorotaxane host–guest linkages between B21C7 and ammonium functionalities (Figure 48b). At high concentrations, the supramolecular cross-linked polymer self-assembled into a metallogel. The metallogel system underwent a reversible gel–sol phase transition upon stimulation with temperature and a KPF_6_/DB18C6 pair (Figure 48c). After adding K (Ⅰ) into the above metallogel system, a gel–sol phase transition was observed. After the addition of enough dibenzo-18-crown-6 to trap K(Ⅰ), the metallogel was re-formed. The cation-responsiveness was attributed to alternate deconstruction and construction of the B21C7 and ammonium linkages due to the alternation between adding and removing K(Ⅰ). These metallogel systems were considered promising candidates for applications in catalysis, separation, absorption, etc.

**Figure 49:**
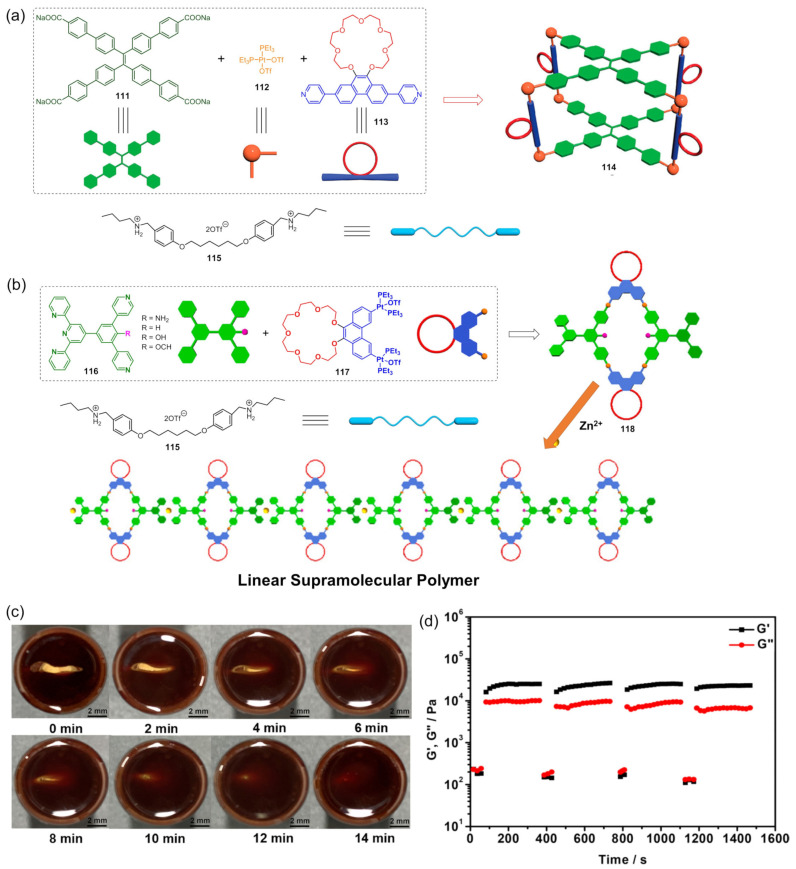
(**a**) Self-assembly of 21C7-Functionalized Metallacage **114** and molecular structure of bis-ammonium salt **115**. (**b**) Cartoon representation of the formation of rhomboidal metallacycles **118**, a linear supramolecular polymer from metallacycles and Zn^2+^, and molecular structure of bis-ammonium salts **115**. (**c**) Photographs of the self-healing process of the supramolecular gel. The gel was cut and left to stand for 2, 4, 6, 8, 10, 12 and 14 min, respectively. (**d**) Gel in continuous step-strain measurements. Reprinted with permission from Refs. [130,131]. Copyright (2018, 2019), American Chemical Society.

Stang et al. reported a tetragonal columnar cage **114** with four appended B21C7 groups in its pillar parts through the metal–ligand interaction-driven self-assembly of a tetraphenylethene (TPE)-based sodium benzoate ligand **111**, cis-Pt(PEt_3_)_2_-(OTf)_2_ **112** and a linear dipyridyl ligand **113** (Figure 49a) [130]. Further introduction of a bis-ammonium salt cross-linker to the as-prepared cage resulted in the formation of a 3D supramolecular polymer network through the host–guest interaction between the B21C7 moiety and the ammonium salts, which formed a jelly-like material at relatively high concentrations. The metallogel showed multiple stimuli-responsiveness and good self-healing properties due to the presence of dynamic metal coordination and host–guest interactions in the whole network structure. A gel–sol state transition was observed visually after heating or adding K(Ⅰ). The heating and the addition of K^+^ weakened the host–guest interactions, which resulted in the collapse of the gel. The gel was restored after a cooling treatment or the addition of 18-crown-6. The gel–sol phase transition was reversible.

**Figure 50:**
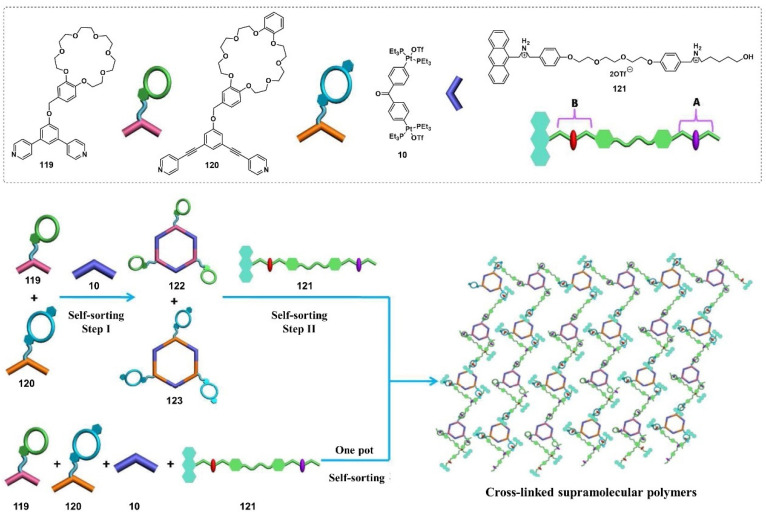
Schematic representation of the construction of cross-linked supramolecular polymers from monomers **119**–**121** and **10** through the stepwise approach (top) and the one-pot approach (bottom). Reprinted with permission from Ref. [132]. Copyright (2015), Royal Society of Chemistry.

Subsequently, Stang’s group designed a novel bicyclic heterometallic cross-linked supramolecular gel with the assistance of three types of orthogonal noncovalent interaction (Figure 49b) [131]. In detail, they first synthesized a ditopic ligand **116** with one end terminating with a terpyridyl group, and the other terminating with two pyridine donor moieties at 120°. The coordination of the 120° dipyridyl group and a Pt(II)-containing monomer **117** gave a rhombic macrocycle **118**. The terminal terpyridyl group in the rhombic organometallic macrocycles was able to coordinate with a Zn(II) ion to form a linear supramolecular polymer. The linear supramolecular polymer was transformed into a cross-linked supramolecular polymer network through a crown ether-based host–guest interaction after adding bis-ammonium salt. At higher concentrations, the cross-linked supramolecular polymer network was able to immobilize solvents to form a supramolecular metallogel. A reversible gel–sol state transition was triggered by various external stimuli such as temperature, K(Ⅰ) and cyclen. The addition of cyclen resulted in the collapse of the metallogel because the cyclen weakened the coordination of the Zn(II) ion and the terpyridyl group, resulting in the disassembly of the linear supramolecular polymer. The metallogel showed interesting self-healing properties. From Figure 49c, it can be seen that the crack on the metallogel gradually disappeared with increasing rest time. Rheological tests were further used to study the self-healing properties. When the strain exceeded 240%, G′ became smaller than G″, indicating a gel–sol phase transition. To further verify the thixotropic property and the reproducibility of this metallogel, large (400%) and small (0.1%) strains were alternately applied to the metallogel system. When the strain was 400%, G″ was larger than G′, suggested that the gel was broken and transformed into a sol. Subsequently, the strain was decreased to 0.1% and the resulting sol was left standing for one minute. The value of G′ and G″ increased immediately and they returned to their original values, suggesting a sol–gel phase transition. This thixotropic process could be repeated many times (Figure 49d). With a similar strategy, Yang et al. designed another cross-linked supramolecular polymer metallogel from four components via a self-sorting strategy [132]. Firstly, two well-defined hexagonal metallacycles **122**–**123** were formed via the coordination-driven assembly of rigid ligands **119**–**120** containing a B21C7 moiety and organoplatinum acceptors **10**. Then, the hexagonal metallacycles were bridged with neutral ditopic guests **121** to give a cross-linked supramolecular polymer network due to the crown ether-based host–guest interaction, after adding bis-ammonium salt (Figure 50). The cross-linked supramolecular polymer network was transformed into stable metallogels at a concentration of 10.0 mM. These metallogels could respond to various external stimuli such as halide, a base and a competitive guest.

**Figure 51:**
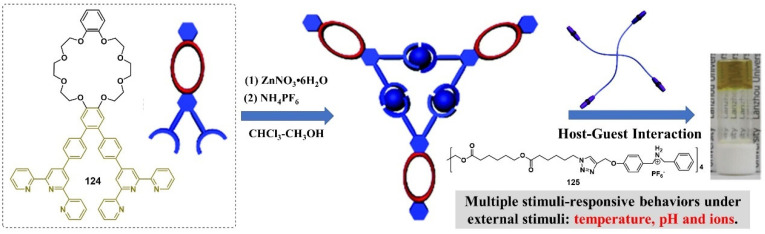
Cartoon representations of a cross-linked three-dimensional supramolecular polymeric network from ligand **124**, Zn^2+^, and bis-ammonium salts **125**. Reprinted with permission from Ref. [133]. Copyright (2016), Royal Society of Chemistry.

In 2016, Bu et al. designed a bis-terpyridine ligand **124** with a dibenzo-24-crown-8 (DB24C8) group [133]. A triangular metal–organic cycle with three DB24C8 groups was formed through a facile coordination reaction of the ligand with Zn(II) ions in a chloroform/methanol mixed solvent, followed by a counter-anion exchange. The triangular metal–organic cycle cross-linked poly(ε-caprolactone)s **125** with four dibenzylammonium salt (DBA)-terminated arms via multiple host–guest interactions between the DB24C8 moiety and DBA. At higher concentrations, the formed network was able to immobilize the solvent molecules to form a stable metallogel (Figure 51). The as-prepared metallogel showed multiple stimuli-responsive behaviors under external stimuli, including temperature, pH and ions (i.e., Cl^−^ and K^+^), due to the presence of dynamic metal–ligand interaction and host–guest interaction.

**Figure 52:**
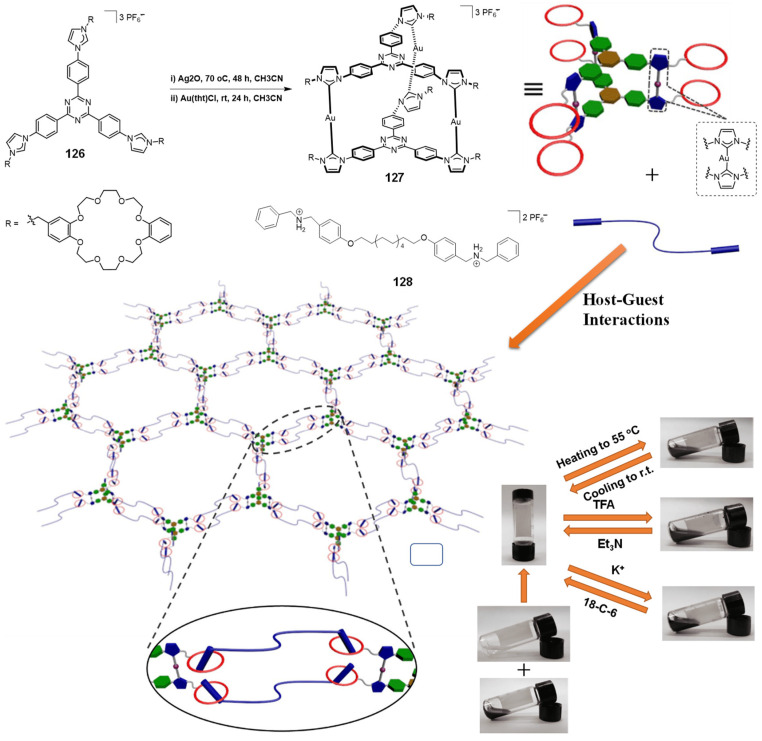
Formation of cross-linked supramolecular polymer network from trinuclear AuI hexacarbene assembly **127** and bis-ammonium salt **128**, and the reversible gel–sol transition of the supramolecular gel. Reprinted with permission from Ref. [134]. Copyright (2021), Elsevier.

Recently, Han et al. designed and synthesized a family of cylinder-like Au(I) trinuclear hexacarbene assemblies [Au_3_(**126**)_2_](PF_6_)_3_ **127** from a trisimidazolium ligand **126,** featuring crown ether groups of different sizes (B15C5, B18C6, B21C7 and DB24C8) at each N-wingtip, via metal–ligand interactions [134]. Supramolecular cross-linked polymer networks were formed when the gold carbene of [Au_3_(**126**)_2_](PF_6_)_3_, appended with DB24C8 as a core, was coupled with bis-ammonium salt **128** as the cross-linker through host–guest interactions between the crown ether groups (DB24C8) and ammonium salts. At higher concentrations, a stable metallogel was formed. Due to the dynamic nature of the host–guest interaction between crown ethers and organic ammonium salts, the resulting metallogel was found to be a stimuli-responsive supramolecular gel. Various external stimuli, including K^+^, temperature and pH value, could induce a reversible gel–sol phase transition (Figure 52). The responsiveness was attributed to dissolution and reformation of the host–guest interactions.

**Figure 53:**
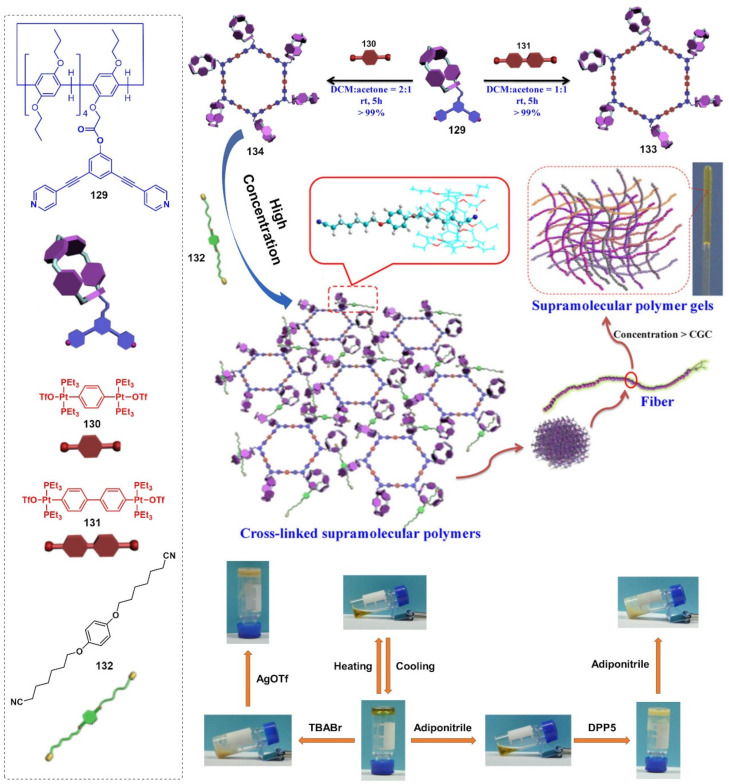
Schematic representation of the hexagonal macrocyclic assembly, the formation of supramolecular polymer gels, and reversible gel–sol transitions of supramolecular polymer gels. Reprinted with permission from Ref. [135]. Copyright (2014), American Chemical Society.

In addition to the combination of crown ether-based host–guest interaction and metal coordination, the pillararene-based host–guest interaction and metal coordination can also be applied to fabricate supramolecular polymer metallogels. In 2014, Yang et al. reported a cross-linked supramolecular metallogel constructed from discrete multi-pillar[5]arene metallacycles and a neutral dinitrile guest via multiple host–guest interactions [135]. In detail, a series of discrete hexakis-pillar[5]arene metallacycles of different sizes were prepared via coordination-driven self-assembly with the assistance of the coordination between 120° monofunctionalized pillar[5]arene dipyridyl ligands **129** and 180° linear di-Pt(II) acceptors **130**–**131** of different lengths. The discrete hexagonal organometallic macrocycles **133** and **134** were bridged by a series of different neutral ditopic guests **132**. At higher concentrations, the cross-linked polymer network was transformed into stable supramolecular gels. The as-prepared metallogels were able to respond to different stimuli, including temperature, halide and competitive guests. A reversible gel–sol phase transition was observed through the addition and removal of adiponitrile. The preferred host–guest interactions between hexakis-pillar[5]arene metallacycles and adiponitrile resulted in disassembly of the cross-linked network, showing a gel–sol transition. Adding excessive 1,4-bis(*n*-propoxy)pillar[5]arene (DPP5, as the competitive host) into the resulting solution reconstructed the gel. In addition, a reversible gel–sol state transformation was triggered by alternately adding bromide and silver ions, accompanied by the disassembly and reassembly of the discrete multi-pillar[5]arene metallacycles (Figure 53).

**Figure 54:**
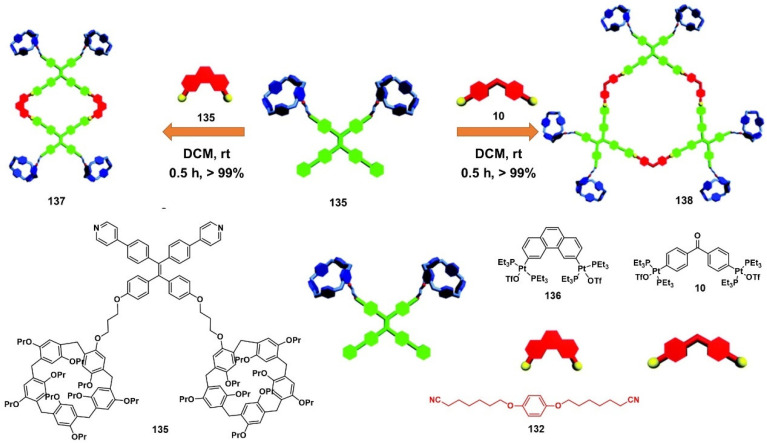
Graphical representation of the self-assembled metallacycles **137** and **138**. Reprinted with permission from Ref. [136]. Copyright (2018), Royal Society of Chemistry.

In 2018, Yang et al. synthesized a dipyridyl ligand **135** with a tetraphenylethylene (TPE) core and two pillar[5]arene units. Rhomboidal **137** and hexagonal metallacycles **138** were developed via coordination with different acceptors **10** and **136 [136].** A self-assembled supramolecular polymer was formed with the introduction of neutral ditopic guests **133** via host–guest recognition. By increasing the concentration of supramolecular polymers, a series of cross-linked supramolecular metallogels were formed with AIE properties. The resulting metallogels exhibited a gel–sol phase transition under stimulation with external stimuli, including temperature, competitive guest molecules and halides, along with “on-off” fluorescence (Figure 54).

**Figure 55:**
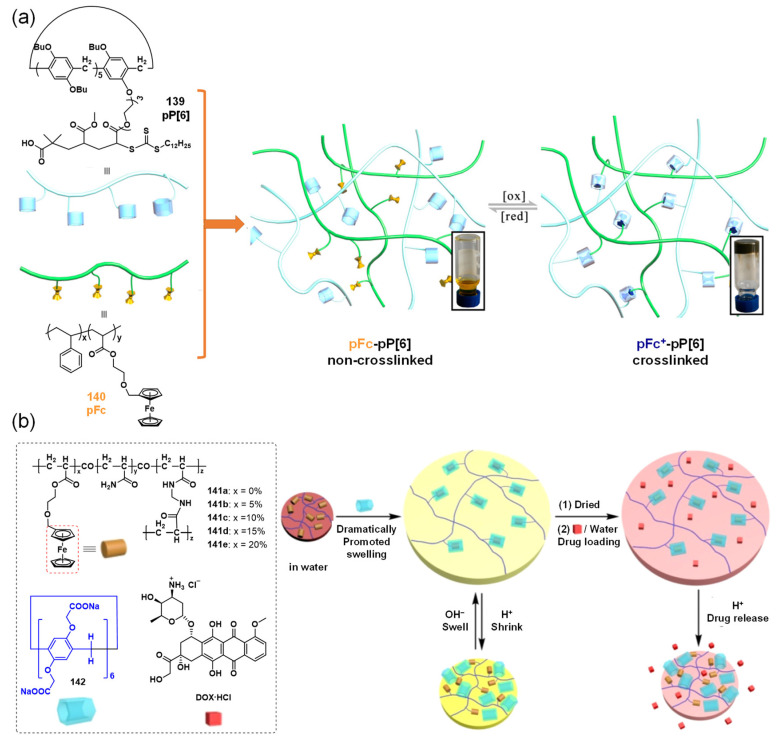
(**a**) Representation of the redox-controllable supramolecular network pFc+-pP [6] based on the functionalized polymers **139** and **140**. (**b**) Illustration of the dramatically promoted swelling of gels via **142**−ferrocene host−guest interactions and subsequent pH-responsive swelling−shrinking transition, and their application in controlled drug (DOX·HCl) release. Reprinted with permission from Refs. [137,138]. Copyright (2015, 2016), American Chemical Society.

In 2015, Wang et al. synthesized two random copolymers with pendant pillar[6]arene groups pP[6] **139** and ferrocene groups pFc **140**, respectively [137]. Simply mixing the two polymers in equal amounts resulted in a free-flowing orange solution. However, mixing a solution of pP[6] and preoxidized pFc^+^ yielded a supramolecular network that immobilized the solvents, and thus, resulted in the formation of a metallogel. A reversible gel–sol phase transition was observed via treatment with external stimuli, including redox stimulus and competing host–guest reagents (Figure 55a). Subsequently, the authors reported a multiple-functional metallohydrogel with a ferrocene moiety on the pendant of the polymer backbone **141** [138]. A much-swollen hydrogel was formed due to the formation of the inclusion complexes between the water-soluble pillar[6]arene (WP6) **142** and ferrocene groups in the hydrogel. The host–guest interaction between WP6 and ferrocene was sensitive to a pH change. When a HCl aqueous solution was added to the swollen **141c·WP6** hydrogel, the gel shrank, which was probably caused by decomplexation of the host−guest complex. The subsequent addition of NaOH aqueous solution resulted in recovery of the swelling property. The redox species (e.g., AgNO_3_/hydrazine hydrate redox pair) also triggered a reversible swelling and shrinking process due to a change in the binding affinity between WP6 and the ferrocenium derivative. Some competitive guests (e.g., N,N′-dimethyl-4,4′-bipyridinium bromide) also induced a responsive process due to the disassembly of the inclusion complex of WP6 and a ferrocene group. In addition, a swelling−shrinking change in the hydrogel, induced by the pH-controlled WP6−ferrocene complexation, gave them potential for application in controlled drug release (Figure 55b).

**Figure 56:**
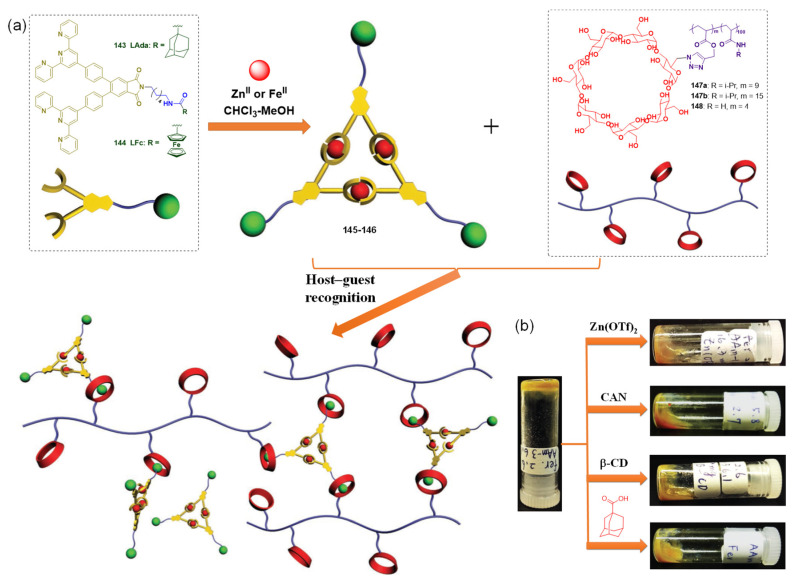
(**a**) Schematic illustration of supramolecular gels constructed through hierarchical self-assembly based on metal–ligand complexation and host–guest recognition. (**b**) Photographs of gel–sol transitions for **145**/**148** triggered by various stimuli. Reprinted with permission from Ref. [139]. Copyright (2018), John Wiley and Sons.

In 2018, Chan et al. synthesized two ditopic terpyridine-based ligands **143** and **144** functionalized with 1-adamantyl (Ada) and ferrocenyl (Fc) groups, respectively [139]. A series of triangular metallocycles **145** and **146** were generated after coupling with Zn(Ⅱ) and Fe(Ⅱ) via a metal–ligand interaction. A host–guest interaction between these triangular metallocycles and β-CD-containing polymers facilitated the formation of polymer networks, which was responsible for the formation of supramolecular metallogels at high concentrations (Figure 56a). The metallogels showed effective stimuli-responsive performance towards cation ions (i.e., Zn(II)) and neutral chemicals (i.e., CAN, β-CD and adamantyl) with a gel–sol phase transition (Figure 56b).

**Figure 57:**
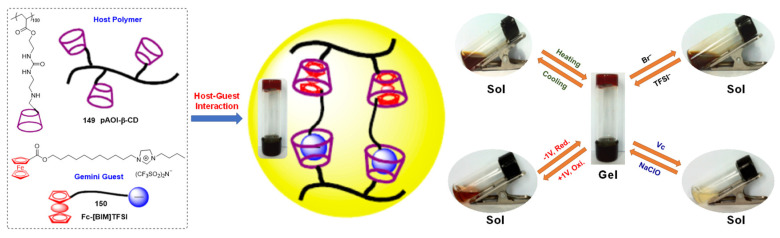
Preparation of ionic liquid gemini guest cross-linked electroactive supramolecular gels and their assembly and disassembly, induced by multi-stimuli. Reprinted with permission from Ref. [140]. Copyright (2014), American Chemical Society.

Yan et al. reported a family of supramolecular metallogels through a host–guest interaction between a host polymer **149** with a β-CD group and an ionic liquid-type asymmetric gemini guest **150** consisting of ferrocene and bis(trifluoromethyl-sulfonyl)imide [140]. A switchable change between the gel and sol state was triggered by a variety of stimuli, including temperature, electrochemical/chemical redox and anion exchange reactions (Figure 57).

## 3. Conclusions and Outlook

Metallogels, with their distinctive dynamic natures and chemo-mechanical behaviors, have been attracting increasing attention in recent decades due to their special optical, electronic, redox or magnetic characteristics. This type of gel material has exhibited great potential for application in the fields of sensing, catalysis, magnetism, drug delivery, optoelectronics, nanoparticle templating, environmental remediation and biological systems. Notably, compared to traditional supramolecular gels, metallogels are able to respond to a wider range of physical and chemical stimuli. To date, various stimuli-responsive metallogel systems have been successfully designed and fabricated to respond to different exogenous stimuli, including temperature, light, sound, mechanical force, magnetic fields, electric fields, pH and chemicals. In this review, we have summarized recent developments in stimuli-responsive supramolecular metallogels with different external stimulus sources, such as chemical-responsive metallogels, physical-responsive metallogels and multiple stimuli-responsive metallogels.

Although a great number of smart stimuli-responsive metallogels have been constructed, many challenges still remain. Firstly, metallogel systems with high mechanical strength and efficient responsive abilities are still rare. Therefore, more efforts should be devoted to developing more efficient metallogelators. Moreover, in-depth insight into the self-assembly and stimuli-responsive mechanisms at the molecular level is required for the discovery of smart stimuli-responsive metallogel systems. Secondly, stimuli-responsive metallic hydrogels with outstanding functionalities are still rarely reported, which limits their further application in biological fields. Some stable, biocompatible and water-soluble organometallic complexes should be incorporated into gelators to fabricate functional hydrogels. Thirdly, although stimuli-responsive metallogels exhibit many promising applications in sensors, catalysis, optoelectronics and environmental remediation, functional stimuli-responsive metallogels are still in their infancy. Further applications in drug delivery and biomedicine need to be further explored [141].

In summary, we have provided a comprehensive overview of the state of the art of stimuli-responsive supramolecular metallogels constructed from organometallic compounds, discrete coordination complexes and coordination polymers. With rapid developments and increasingly exceptional research in supramolecular chemistry, material sciences and organometallic chemistry, we firmly believe that the research area of stimuli-responsive metallogels will continue to blossom in the future.

## Data Availability

The data that support the findings of this study are contained within the article. More information is available on request from the corresponding author.
